# Socioeconomic inequalities in prevalence and development of multimorbidity across adulthood: A longitudinal analysis of the MRC 1946 National Survey of Health and Development in the UK

**DOI:** 10.1371/journal.pmed.1003775

**Published:** 2021-09-14

**Authors:** Amal R. Khanolkar, Nishi Chaturvedi, Valerie Kuan, Daniel Davis, Alun Hughes, Marcus Richards, David Bann, Praveetha Patalay

**Affiliations:** 1 MRC Unit for Lifelong Health and Ageing at UCL, London, United Kingdom; 2 Department of Global Public Health, Karolinska Institutet, Stockholm, Sweden; 3 Institute of Health Informatics, UCL, London, United Kingdom; 4 Institute of Cardiovascular Science, UCL, London, United Kingdom; 5 Centre for Longitudinal Studies, UCL, London, United Kingdom; Harvard Medical School, UNITED STATES

## Abstract

**Background:**

We aimed to estimate multimorbidity trajectories and quantify socioeconomic inequalities based on childhood and adulthood socioeconomic position (SEP) in the risks and rates of multimorbidity accumulation across adulthood.

**Methods and findings:**

Participants from the UK 1946 National Survey of Health and Development (NSHD) birth cohort study who attended the age 36 years assessment in 1982 and any one of the follow-up assessments at ages 43, 53, 63, and 69 years (*N =* 3,723, 51% males). Information on 18 health conditions was based on a combination of self-report, biomarkers, health records, and prescribed medications. We estimated multimorbidity trajectories and delineated socioeconomic inequalities (based on childhood and adulthood social class and highest education) in multimorbidity at each age and in longitudinal trajectories.

Multimorbidity increased with age (0.7 conditions at 36 years to 3.7 at 69 years). Multimorbidity accumulation was nonlinear, accelerating with age at the rate of 0.08 conditions/year (95% CI 0.07 to 0.09, *p* < 0.001) at 36 to 43 years to 0.19 conditions/year (95% CI 0.18 to 0.20, *p* < 0.001) at 63 to 69 years. At all ages, the most socioeconomically disadvantaged had 1.2 to 1.4 times greater number of conditions on average compared to the most advantaged. The most disadvantaged by each socioeconomic indicator experienced an additional 0.39 conditions (childhood social class), 0.83 (adult social class), and 1.08 conditions (adult education) at age 69 years, independent of all other socioeconomic indicators. Adverse adulthood SEP was associated with more rapid accumulation of multimorbidity, resulting in 0.49 excess conditions in partly/unskilled compared to professional/intermediate individuals between 63 and 69 years. Disadvantaged childhood social class, independently of adulthood SEP, was associated with accelerated multimorbidity trajectories from age 53 years onwards.

Study limitations include that the NSHD cohort is composed of individuals of white European heritage only, and findings may not be generalizable to the non-white British population of the same generation and did not account for other important dimensions of SEP such as income and wealth.

**Conclusions:**

In this study, we found that socioeconomically disadvantaged individuals have earlier onset and more rapid accumulation of multimorbidity resulting in widening inequalities into old age, with independent contributions from both childhood and adulthood SEP.

## Abstract 2

Amal Khanolkar and co-workers study associations between multimorbidity and socioeconomic position in the UK.

## Author summary

**Why was this study done?:**

- Multimorbidity—2 or more chronic conditions in an individual—has several consequences including reduced quality of life, reduced life expectancy, and complex healthcare needs.
- Socioeconomic disadvantage is associated with both earlier onset (at younger ages) and greater burden of multimorbidity. However, less is known about how multimorbidity develops across adulthood and into older ages in the same individuals and differences in its development by childhood and adulthood socioeconomic position (SEP).

**What did the researchers do and find?:**

- Longitudinal multimorbidity trajectories were estimated between ages 36 and 69 years, and whether these trajectories differed by childhood (father’s social class) and adulthood SEP (participant’s own social class and educational level).
- We found that throughout follow-up, the most socioeconomically disadvantaged had 1.2 to 1.4 times greater number of health conditions on average compared to the most advantaged.
- On average, multimorbidity increased across adulthood and into older ages in both socioeconomically disadvantaged and advantaged groups. However, the rate at which multimorbidity increased across adulthood differed by SEP, with those from the most disadvantaged backgrounds having worse trajectories. In other words, adverse SEP was associated with more rapid accumulation of multimorbidity.
- Childhood and adulthood SEP were independently associated with multimorbidity across adulthood. This indicates that childhood socioeconomic disadvantage is still evident in later life over and above adulthood socioeconomic circumstances.

**What do these findings mean?:**

- The independent and long-lasting shadow of childhood SEP on the experience and the rate of accumulation of multimorbidities in later life highlight the need for interventions early in life to minimise inequalities in later life health.
- This calls for population-based interventions in early life and through the lifecourse to reduce the impact of childhood and adulthood inequalities, along with better access and delivery of healthcare for the more vulnerable to help reduce the burden of multimorbidity.

## Introduction

The management of long-term chronic health conditions poses a serious challenge for public healthcare systems worldwide [1,2]. Conditions associated with unhealthy lifestyles, such as obesity, hypertension, diabetes, and coronary heart disease are increasingly common, while greater life expectancies mean individuals live longer with chronic disease. These conditions rarely occur in isolation, hence multimorbidity—2 or more chronic conditions in an individual—is a rising consequence [3]. Multimorbidity is associated with poorer quality of life, decreased work productivity, increased use of ambulatory and inpatient healthcare, polypharmacy, increased functional limitations, and mortality [4–9]. Socioeconomic inequalities in health are well established, and socioeconomic disadvantage is associated with both a greater burden of multimorbidity and earlier onset [2,10–22].

Most studies on the epidemiology of multimorbidity, and specifically those that have investigated socioeconomic inequalities, have largely been cross-sectional in design [2,4,12,13,15,17,23]. The few longitudinal studies vary substantially in design (cohorts following same individuals and serial cross-sectional surveys) and follow-up time (from 5 to 25 years) [14,16,24–26], and to our knowledge, no study has examined the longitudinal development of multimorbidity, including rate of accumulation, in the same individuals and both sexes across adulthood. Additionally, the longitudinal effects of early and later life socioeconomic position (SEP) on prevalence and development of multimorbidity is not known.

Independent effects of socioeconomic disadvantage in childhood on multimorbidity development across the lifecourse are not known. This is an important gap in knowledge, as socioeconomic disadvantage in childhood has lasting impacts on health, independently of adult socioeconomic circumstances [27,28]. Excluding childhood socioeconomic circumstances might underestimate the extent of the impact of socioeconomic disadvantage across the lifecourse.

Further, few studies to date have investigated multimorbidity development in the same individuals across the lifecourse, and no studies investigated its association with lifetime SEP, as this requires individual-level population-based longitudinal data over several decades. Given ageing-related processes and health deterioration, it is expected that the rate of multimorbidity accumulation will accelerate over the lifecourse. Any socioeconomic differentials in the rate of disorder accumulation are important to establish as they help uncover whether the inequalities observed at older ages are driven by earlier onset, faster accumulation, or both.

This study examines multimorbidity development across adulthood and into older ages and investigates in detail socioeconomic inequalities in multimorbidity trajectories in a prospective nationally representative birth cohort study. We estimated multimorbidity development in the same individuals over time and, importantly, estimate the independent effects of childhood and adulthood socioeconomic circumstances on multimorbidity prevalence and rate of accumulation.

## Methods

### Study participants and setting

This study analysed data from participants in the MRC 1946 National Survey of Health and Development (NSHD) [29]. The NSHD includes a socially stratified and nationally representative sample of 5,362 participants born in 1 week in March 1946 in England, Scotland, and Wales and recruited at birth. Extensive social, anthropometric, medical, lifestyle, and biological data were collected either during home visits or at clinical research centres in later years by professional interviewers and research nurses. More information on the study is available from the study’s website: https://www.nshd.mrc.ac.uk/. Each sweep of data collection has the required ethics approvals, and study members provide their consent for each assessment sweep.

The analysis sample for this prospective longitudinal study included participants who attended the age 36 years assessment in 1982 and any one of the follow-up assessments at ages 43 in 1989, 53 in 1999, 63 in 2009, and 69 years in 2015 (total *N =* 3,723, including 1,843 women [49.5%]). Participants lost to follow-up (due to death or emigration) between ages 36 and 63 were excluded from the study sample.

Attrition: Of the original 5,362 cohort participants, 3,723 participants were included in this analysis. By age 63 years, 718 (13.4%) had died, 594 (11.1%) had previously withdrawn from the study, 567 (10.6%) lived abroad, and 320 (5.9%) had been untraceable for more than 10 years.

### Conditions

Data for 18 selected common chronic conditions on which consistent and reliable information was collected across the adulthood sweeps are the focus of this study [30]. These included diabetes, dyslipidaemia, hypertension, obesity, coronary heart disease, stroke, cancer, anaemia, respiratory disorders, kidney disorders, gastrointestinal disorders, skin disorders, osteoarthritis, rheumatoid arthritis, Parkinson disease, epilepsy, depression, and psychosis. Including conditions with consistently collected data across the 5 sweeps would aid in estimating total multimorbidity score at each time point. Data sources, sweeps at which information is available and definitions for each of the 18 conditions, are listed in Table A in S1 File.

### Chronicity

We used the Delphi technique [31]—a structured communication tool—to give an informed consensus on which conditions would be considered chronic (i.e., once a participant was diagnosed with a condition then the participant would be considered to have the condition throughout follow-up). The Delphi team consisted of 10 clinicians (of ranging specialities including general practice, cardiovascular, geriatrics, and respiratory medicine) who received the same form that listed the 18 conditions, how they were measured, and a column for them to indicate which ones they would consider chronic (i.e., to be carried forward; Table B in S1 File). Each clinician was requested to indicate their decision along with a brief explanation supporting their decision. All responses were collated and examined in an Excel sheet. We chose the majority decision for each condition on whether it should be considered chronic or not.

### Multimorbidity

All 18 conditions were coded as binary variables indicating whether a participant had a condition or not at each sweep. The multimorbidity score in this paper is defined as the unweighted sum of the number of conditions (out of 18 conditions) accumulated by an individual [2,14].

For those conditions considered chronic (like diabetes or hypertension), a participant was coded to have the condition throughout follow-up once diagnosed (Table A in S1 File). For all participants, we calculated a multimorbidity score at each age (i.e., across the 5 age sweeps), which indicated the number of conditions at each time point, and this multimorbidity score was the main outcome.

### Socioeconomic position (SEP)

Childhood SEP was based on the father’s occupational social class, reported at age 11. We used 2 measures of SEP in adulthood: educational level attained at age 33 and occupational social class ascertained at age 43. Occupational social class was coded into 4 categories: (1) Professional and intermediate (reference group in analysis); (2) Nonmanual; (3) Manual; and (4) Partly skilled/unskilled. Educational levels were ascertained by the Burnham classification. The original 9 categories were collapsed into 3 groups for analysis: (1) None or vocational courses; (2) School leaving certificate (GCE “O” and “A” levels); and (3) Degree level and higher (university education, reference group).

### Statistical analysis

We used frequencies, percentages (prevalence of each condition at all age sweeps for the full study sample), cross-tabulations (mean differences in multimorbidity score across the 5 age sweeps by socioeconomic indicators), and graphical display for descriptive analysis. To visualise prevalence and associations between the conditions that constituted the multimorbidity score at each age, networks were constructed using the R package “igraph” (conditions represented by nodes with sizes proportional to prevalence). These networks effectively illustrate the changing makeup of the total multimorbidity score at each age. Partial correlations between each of the 18 conditions are represented as “edges” in networks (networks only included those edges with partial correlations with absolute values >0.05 and *p*-values <0.05). The width of an edge was proportional to the strength of the partial correlation between 2 conditions (with positive partial correlations >0.05 represented by grey edges and negative correlations <−0.05 shown in red).

Socioeconomic patterning in multimorbidity at each of the 5 age sweeps was analysed using multivariable linear regression. We first ran models to test individual associations between each of the 3 indicators and the multimorbidity score. The fourth model was mutually adjusted for all 3 socioeconomic indicators in order to assess each of their associations independent of other indicators. All models were adjusted for sex.

Longitudinal change in multimorbidity trajectories were analysed using linear spline mixed-effects modelling. This accounts for repeated measurements (or clustering) of multimorbidity scores within individuals over time and individual-level deviations from an overall average trajectory, with a nonlinear specification of the average trajectory using linear splines [32]. Given the data structure—cohort members are of the same age at each time point—knots were assigned at ages 43, 53, 63, and 69 years giving 4 splines corresponding to ages 36 to 43, 43 to 53, 53 to 63, and 63 to 69 years. The model with a random intercept plus random slopes for the individual spline coefficients had a superior fit and was used in all subsequent modelling. This breaks up the average change in multimorbidity score into separate slopes for each of the 4 spline variables representing an age band (each slope coefficient indicating rate of change in multimorbidity score per year in each period). We used linear spline mixed-effects modelling to first estimate the population-average multimorbidity trajectory for the full sample and by sex. The model for multimorbidity trajectories by sex included interaction terms between sex and the spline variables for ages 36 to 43, 43 to 53, and 53 to 63 years. We then ran 4 models, each one having a different socioeconomic exposure of interest plus sex: Model 1: childhood social class; Model 2: adulthood social class; Model 3: adulthood educational level; and Model 4: mutual adjustment for all 3 socioeconomic indicators.

To examine whether the slope of multimorbidity trajectories varied by social class categories over time within each of the 4 spline variables (i.e., whether inequalities in the rate of multimorbidity accumulation changed over time), we tested for interactions between childhood or adulthood social class and the individual spline variables. Only those interactions terms that were significant (*p* < 0.05) were subsequently included in the model. The model for adulthood social class included interactions with all 4 spline variables (as all 4 interactions were significant, *p* < 0.05), whereas the one for childhood social class included interactions with the ages 53 to 63 years and 63 to 69-years’ variables only. These models were mutually adjusted for other socioeconomic indicators. Interactions were significant, and multimorbidity trajectories were plotted at the group level (childhood and adulthood social class separately) for visualisation based on model 4 above. Interactions between spline variables and education were not significant and are not shown.

Missing data across the 5 age sweeps were addressed using multiple imputation assuming missing at random (MAR) using 25 imputed datasets. The MAR mechanism implies that systematic differences between the missing values and the observed values can be explained by observed data [33], which is a plausible assumption in the British birth cohorts given the rich data available from birth [34]. Additionally, the main purpose was to impute missing data in health conditions across the 5 sweeps. The imputed data are more likely to be closer to the true values (i.e., predicted with greater precision), as many of the health conditions are correlated longitudinally further strengthening the imputation model. Information on the 3 socioeconomic indicators was available for more >94% of study participants. Frequencies and distributions of nonimputed and imputed variables were largely similar for most conditions (Table C in S1 File). All analyses including the descriptive analysis were run on the imputed sample (*N =* 3,723). All analyses were conducted using Stata version 15 (StataCorp LP, College Station, Texas). A completed STROBE checklist is provided as a separate supplementary file (S1 STROBE Checklist). Analyses were prespecified, but no written analysis plan is available.

## Results

**Fig 1:**
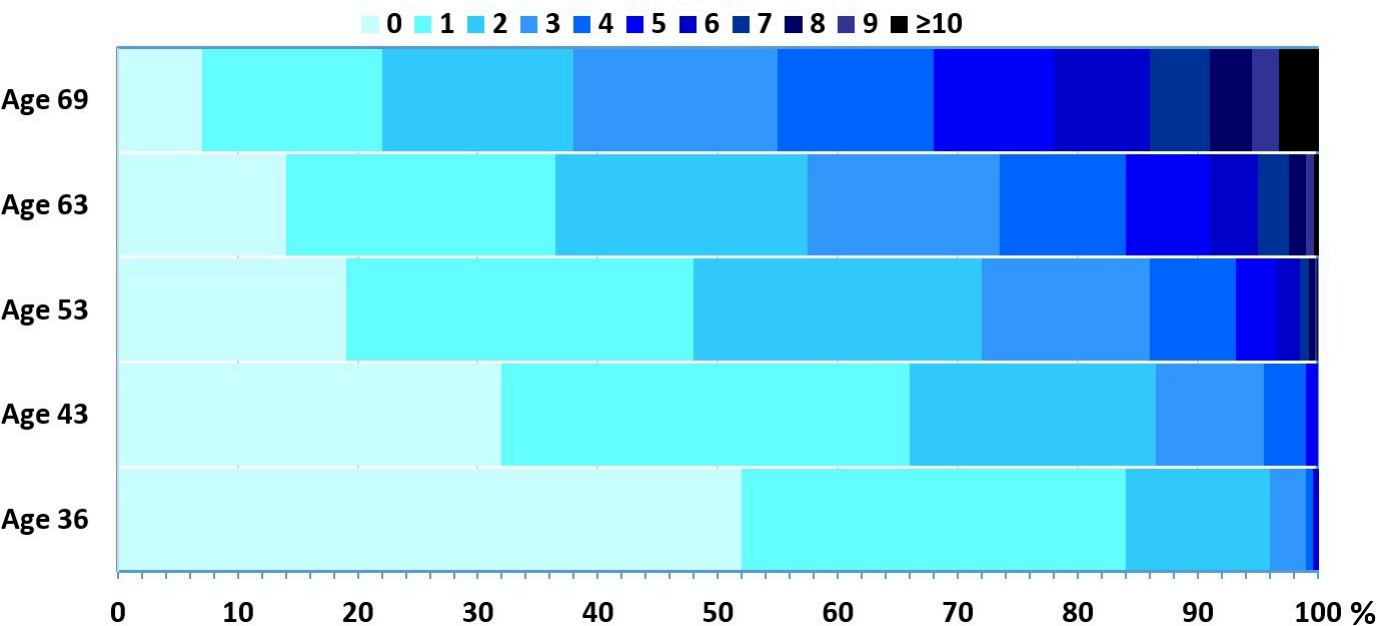
Percentage of participants with increasing levels of multimorbidity (based on 18 common conditions) at each age sweep in 3,723 participants from the 1946 MRC NSHD. NSHD, National Survey of Health and Development.

**Fig 2:**
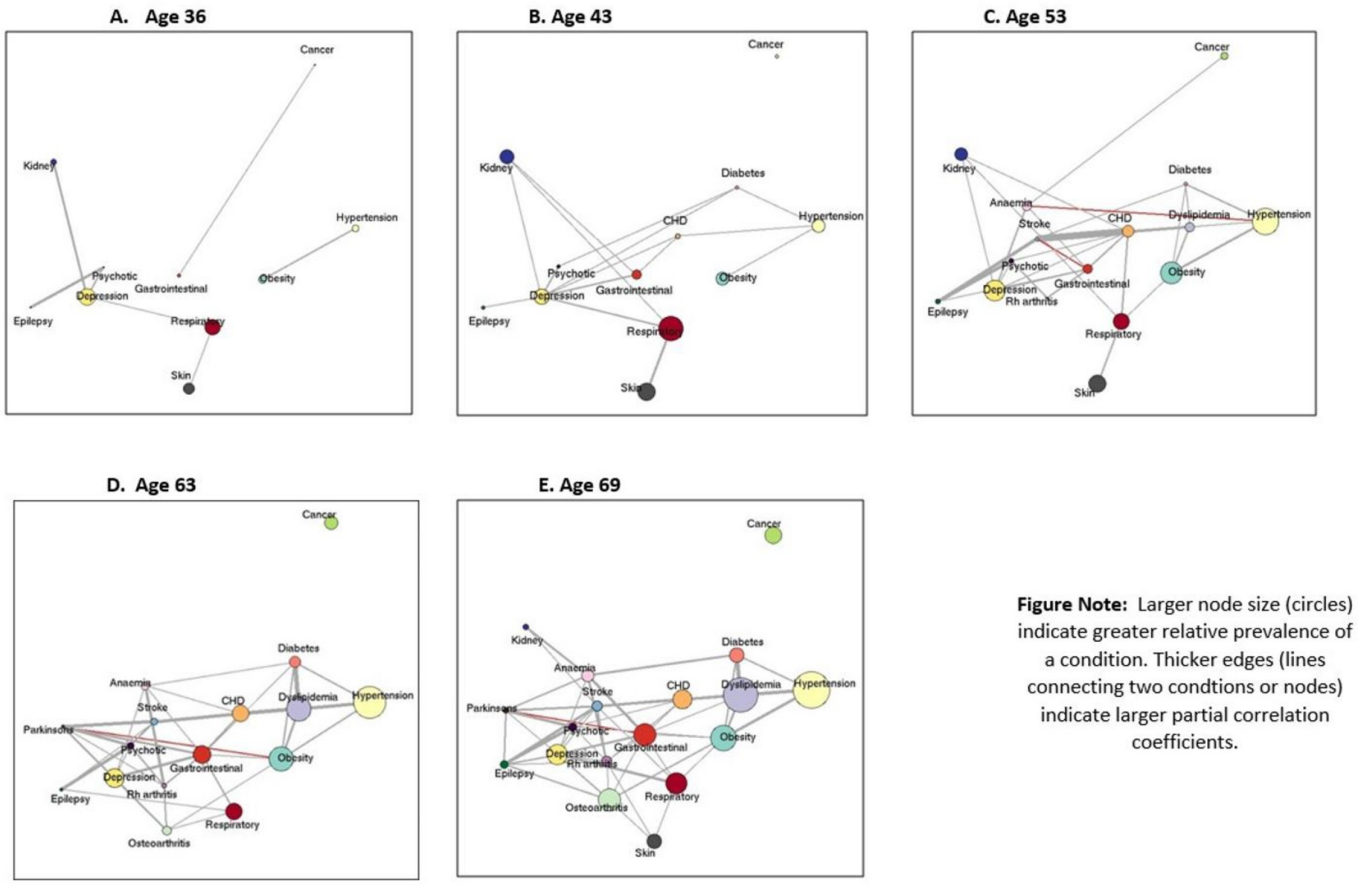
Network analysis illustrating relative prevalence (node size) of conditions and adjusted associations (edges) at each age in 3,723 participants from the 1946 MRC NSHD. CHD, coronary heart disease; NSHD, National Survey of Health and Development.

Table 1 shows the prevalence of the 18 conditions across the 5 ages. We found that the prevalence of most conditions increased with age (for example, obesity and hypertension increased from 7% and 6% at age 36 to 34% and 52% at age 69 years, respectively). The proportion with no conditions decreased progressively from 52% at age 36 to 7% at age 69 years (Fig 1). Fig 2 displays results from network analysis with node size representing relative prevalence of a condition and edges representing associations between conditions at each of the 5 ages. These networks illustrate both the relative increase in prevalence of disorders over time and the greater interconnectivity between conditions with age. For example, prevalence of hypertension increases with age, and its association with dyslipidaemia gets stronger over time. In contrast, although more individuals have cancer over time, cancer is not independently associated with experiencing any of the other conditions at most ages (indicated by no edges between cancer and other conditions). Similarly, skin conditions are independent of most conditions over time, apart from respiratory conditions.

**Table 1:** Prevalence (%) of conditions and multimorbidity at each of the 5 age sweeps in 3,723 participants from the 1946 MRC NSHD.

	Age (prevalence [95% CI])				
Condition	Age 36	Age 43	Age 53	Age 63	Age 69
**Obesity**	6.9 (6–7.7)	12.8 (11.6–13.9)	25.0 (23.2–26.6)	32.2 (30–34.4)	34.4 (32–36.7)
**Hypertension**	6.3 (5.4–7.1)	13.3 (12.1–14.5)	32.4 (30.7–34.1)	44.8 (42.9–46.6)	52.0 (50–53.9)
**Dyslipidaemia**	N.A.	N.A.	9.1 (7.9–10.2)	32.2 (30–34.3)	47.8 (45.4–50)
**Diabetes**	N.A.	1.5 (1–1.9)	3.2 (2.5–3.8)	10.7 (9.4–11.9)	16.3 (14.8–17.7)
**CHD**	N.A.	3.8 (3.1–4.5)	11.6 (10.2–12.9)	20.2 (18.5–22)	22.8 (20.9–24.5)
**Stroke**	N.A.	N.A.	2.5 (1.4–3.6)	6.4 (4.7–8.2)	10.3 (8.6–11.9)
**Osteoarthritis**	N.A.	N.A.	N.A.	9.4 (7.8–10.9)	27.5 (25.1–29.8)
**Rheumatoid arthritis**	N.A.	N.A.	1.3 (0.5–2.2)	4.2 (2.5–5.9)	9.7 (7.3–12)
**Anaemia**	N.A.	N.A.	9.0 (7.8–10.2)	6.8 (5.2–8.5)	12.5 (10.4–14.6)
**Skin disorders**	10.8 (9.7–11.8)	18.6 (17.3–20)	19.1 (17.6–20.5)	N.A.	16 (14.1–18)
**Respiratory disorders**	16.1 (14.8–17.4)	28.4 (26.8–30)	17.5 (15.9–19)	20.4 (18.7–22.1)	25.6 (23.7–27.5)
**Gastrointestinal disorders**	2.3 (1.6–2.8)	8.9 (7.8–9.8)	8.0 (6.7–9.1)	21.0 (18.6–22.7)	28.3 (25.1–21.5)
**Kidney disorders**	4.9 (4.2–5.6)	13.8 (12.6–14.9)	13.3 (11.8–14.8)	N.A.	5.0 (2.8–7.2)
**Parkinson disease**	N.A.	N.A.	N.A.	2.6 (1.2–4)	5.5 (3.8–7.1)
**Cancer**	0.6 (0.3–0.8)	1.7 (1.3–2.1)	5.6 (4.8–6.3)	14.2 (13.1–15.3)	19.5 (18.3–20.9)
**Epilepsy**	1.5 (0.9–1.9)	2.1 (1.4–2.7)	4.2 (2.9–5.4)	3.0 (2.1–4)	8.3 (6.7–9.9)
**Depression**	18.4 (16.9–19.7)	16.4 (15.2–17.7)	23.0 (21.3–24.7)	23.4 (21.7–25.1)	24.5 (22.7–26.9)
**Psychotic disorders**	0.9 (0.5–1.4)	2.0 (1.4–2.7)	3.8 (2.6–5.1)	6.0 (4.4–7.6)	8.0 (6.2–9.8)
**Number of conditions (mean)**	0.7 (0.6–0.7)	1.2 (1.1–1.3)	1.9 (1.8–2.0)	2.6 (2.5–2.7)	3.7 (3.6–3.9)

N.A., not applicable—indicates that data for that condition were not collected at the particular age.

CHD, coronary heart disease; NSHD, National Survey of Health and Development.

**Fig 3:**
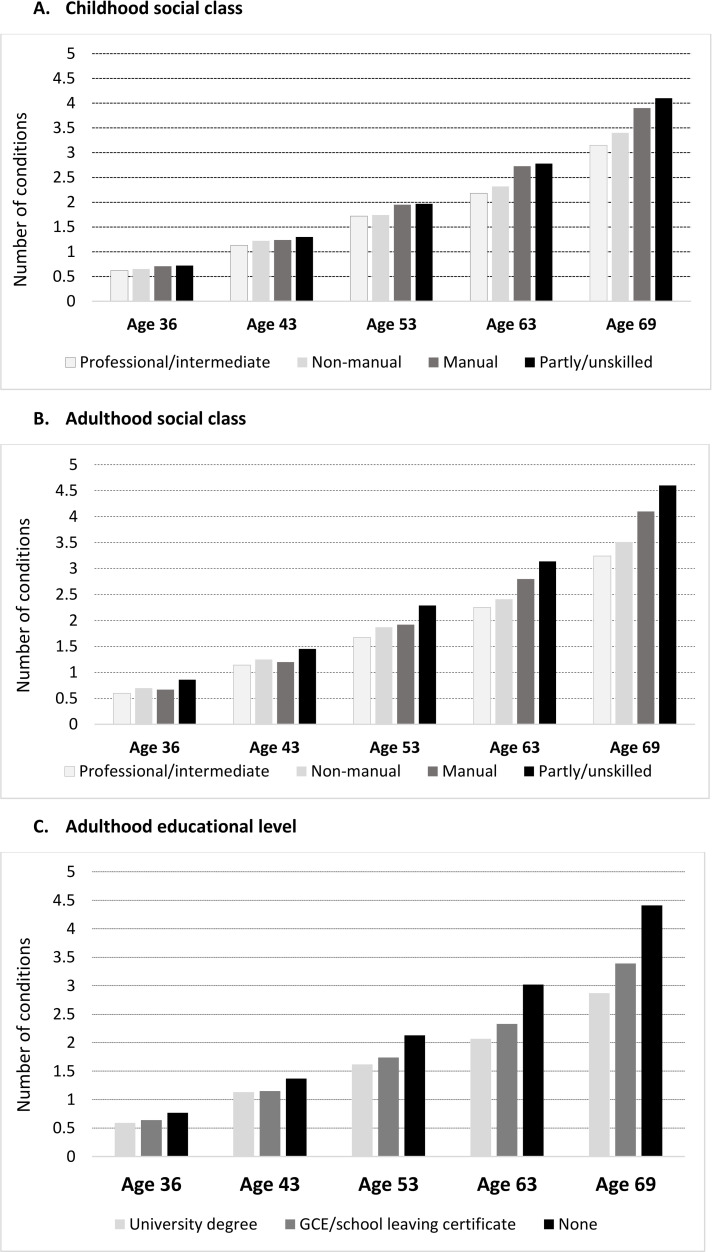
Mean number of morbidities at each age by socioeconomic indicators in 3,723 participants from the 1946 MRC NSHD. GCE, General Certificate of Education; NSHD, National Survey of Health and Development.

We found that, at each age sweep, socioeconomic disadvantage was associated with higher multimorbidity scores (Fig 3, Table 2, and Table F in S1 File). The mean difference in number of conditions between the most and least disadvantaged was observed with childhood and adulthood social class, and with educational level at all ages, and got progressively larger with age. At all ages, the most socioeconomically disadvantaged had a greater number of conditions on average compared to the most advantaged with the largest difference observed at age 69 years with 1.4 more conditions (for instance, 0.60 versus 0.85 at age 36, 1.67 versus 2.28 at age 53, and 3.24 versus 4.59 at age 69 years for professional/intermediate versus partly skilled/unskilled adulthood social class, respectively; Table F in S1 File). Compared with the most advantaged group (professional/intermediate), partly skilled/unskilled individuals had progressively higher multimorbidity scores across adulthood (Table 2; age 36 years: 0.18 [0.08 to 0.28], age 69 years: 0.83 [045 to 1.20]; and Fig A in S1 File). Socioeconomic disadvantage in childhood and in adulthood were independently associated with higher levels of multimorbidity at ages 63 and 69 years (Table 2; 0.39 [0.06 to 0.72] for partly skilled/unskilled childhood social class and 1.08 [0.67 to 1.49] for no education age 69). The most socioeconomically disadvantaged by each measure of SEP, adjusted for all other measures of SEP, experienced an additional 0.39 (childhood social class), 0.83 (adult social class), and 1.08 conditions (adult education) at age 69 years (Table 2).

**Table 2:** Results from linear regression modelling assessing associations between SEP and multimorbidity (unweighted sum of the number of health conditions) in 3,723 participants from the 1946 MRC NSHD.

	Change in multimorbidity score^a^									
	Age 36		Age 43		Age 53		Age 63		Age 69	
Covariates	β	95% CI	β	95% CI	β	95% CI	β	95% CI	β	95% CI
**Childhood social class**										
Professional/intermediate	Ref		Ref		Ref		Ref		Ref	
Skilled nonmanual	0.03	−0.07,0.12	0.08	−0.05,0.21	−0.01	−0.18,0.17	0.11	−0.13,0.35	0.18	−0.13,0.50
Skilled manual	0.05	−0.04,0.13	0.05	−0.07,0.16	0.09	−0.07,0.24	**0.32**	**0.12,0.52**	**0.39**	**0.10,0.68**
Partly skilled/unskilled	0.03	−0.06,0.12	0.07	−0.06,0.20	0.04	−0.14,0.22	**0.25**	**0.00,0.50**	**0.39**	**0.06,0.72**
**Adulthood social class**										
Professional/intermediate	Ref		Ref		Ref		Ref		Ref	
Skilled nonmanual	0.04	−0.04,0.13	0.03	−0.09,0.15	0.12	−0.04,0.27	0.05	−0.15,0.25	0.11	−0.17,0.39
Skilled manual	0.05	−0.04,0.15	0.03	−0.10,0.16	0.13	−0.05,0.31	**0.24**	**0.02,0.46**	**0.33**	**0.03,0.63**
Partly skilled/unskilled	**0.18**	**0.08,0.28**	**0.17**	**0.03,0.32**	**0.43**	**0.24,0.62**	**0.54**	**0.28,0.80**	**0.83**	**0.45,1.20**
**Educational attainment**										
University degree	Ref				Ref		Ref		Ref	
GCE/school leaving certificate	−0.01	−0.11,0.09	−0.05	−0.18,0.09	0.02	−0.18,0.22	0.14	−0.10,0.38	**0.35**	**0.03,0.67**
None	0.06	−0.07,0.18	0.13	−0.04,0.30	**0.28**	**0.04,0.53**	**0.62**	**0.32,0.91**	**1.08**	**0.67,1.49**
**Sex**										
Men	Ref		Ref		Ref		Ref		Ref	
Women	**0.09**	**0.02,0.15**	**0.15**	**0.06,0.24**	0.06	−0.07,0.18	−0.11	−0.27,0.06	**−0.24**	**−0.46,−0.03**

^a^All models adjusted for sex; coefficients in bold indicate 95% confidence intervals that do not include zero.

GCE, General Certificate of Education; NSHD, National Survey of Health and Development; SEP, socioeconomic position.

**Fig 4:**
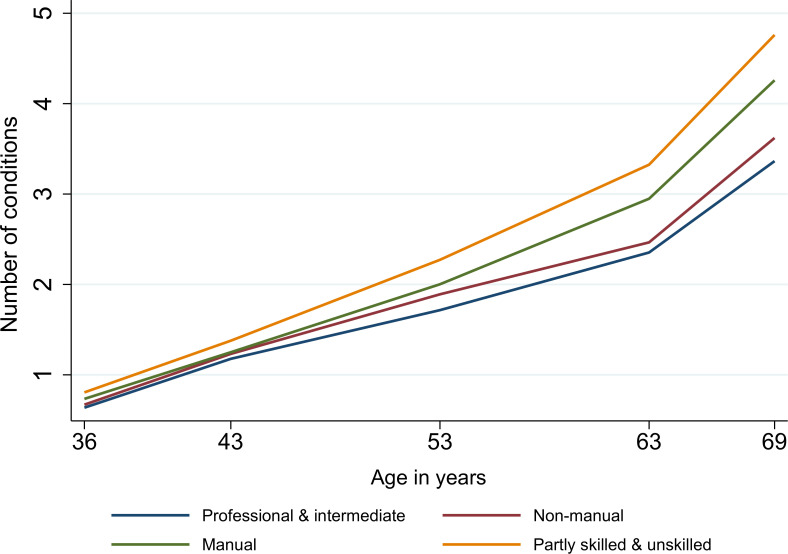
Socioeconomic inequalities in multimorbidity trajectories by adulthood social class in 3,723 participants from the 1946 MRC NSHD. Note: Fig 4 is based on Model 3 in Table G and adjusted for sex, childhood social class, and educational level. NSHD, National Survey of Health and Development.

**Fig 5:**
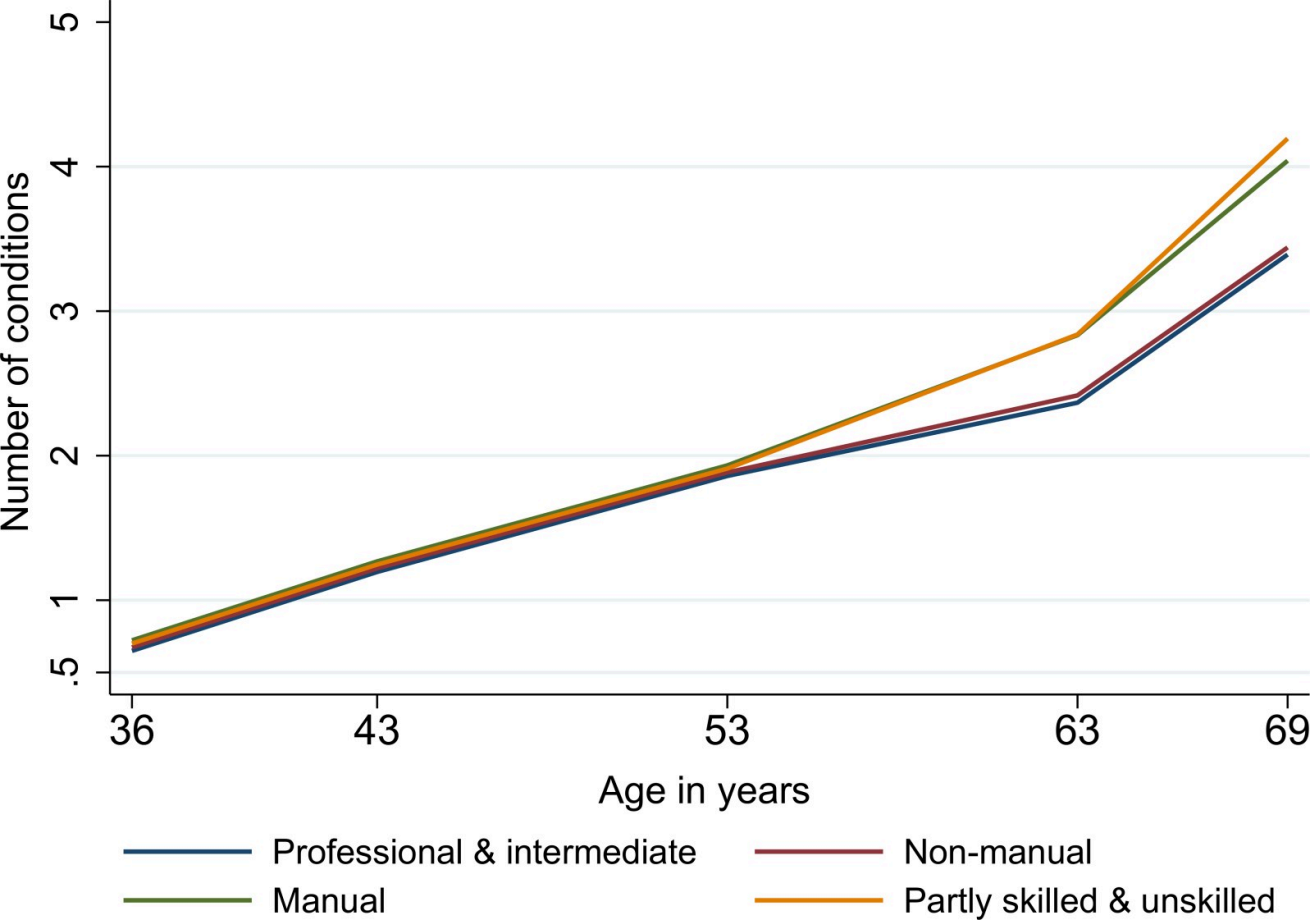
Socioeconomic inequalities in multimorbidity trajectories by childhood social class in 3,723 participants from the 1946 MRC NSHD. Note: Fig 5 is based on Model 2 in Table G and adjusted for sex, adulthood social class, and educational level. NSHD, National Survey of Health and Development.

Overall, multimorbidity increased steadily over adulthood with an accelerated accumulation in older ages [from 0.08 (95% CI 0.07 to 0.09) conditions/year between ages 36 and 43 years to 0.19 (0.18 to 0.20) conditions/year between ages 63 and 69 years (linear spline mixed-effects models, Model 1; Table G in S1 File and Fig B in S1 File)]. Women had slightly higher rates of multimorbidity than men between ages 36 and 53 years, after which there were no further differences in rates by sex (Fig C in S1 File). Further, we observed stark socioeconomic inequalities in multimorbidity trajectories, which increased and widened progressively with age. Between ages 43 to 69 years, disadvantaged adulthood social class groups like manual and partly skilled/unskilled individuals experienced greater acceleration of multimorbidity trajectories (Model 3; Table G in S1 File and Fig 4). For instance, compared to professional/intermediate individuals, manual workers experienced an excess of 0.18 conditions and partly skilled/unskilled individuals 0.31 conditions between ages 36 and 43 years, which increased to 0.30 conditions and 0.49 conditions, respectively between 63 and 69 years (Model 3; Table G in S1 File). The independent (of adult SEP) disparities in multimorbidity trajectories by childhood social class emerged at age 53 years and widened over the remaining period of follow-up (Model 2; Table G in S1 File and Fig 5). In this case, rather than a graded effect by childhood social class, professional and nonmanual childhood social classes both followed a more favourable trajectory than manual and unskilled social classes.

## Discussion

Through following the same individuals in the longest running British birth cohort, we report several novel findings. Firstly, we show that multimorbidity accumulation accelerates with age. Secondly, we found that childhood and adulthood SEP, and achieved education, were each independently associated with an increased risk of multimorbidity burden at each age. Thirdly, we demonstrate that adverse SEP is associated with a more rapid acceleration of multimorbidity accumulation, providing strong evidence for widening inequalities with age. In sum, socioeconomically disadvantaged individuals have both earlier onset and more rapid accumulation of multimorbidity, with independent contributions from both childhood and adulthood SEP.

The few longitudinal studies that have examined associations between SEP and multimorbidity found that low income, low education, low occupational position, and greater area deprivation were associated with increased risk for multimorbidity [10,16,21,22,35,36]. In addition to a shorter follow-up time, previous studies have focused on certain conditions [35] (like cardiometabolic diseases) or analysed disease-specific trajectories [36]. The Whitehall and Twenty-07 studies both examined socioeconomic inequalities in multimorbidity development in the United Kingdom, but the former was restricted to participants aged over 50 in a specific nonpopulation representative sample, while the latter reported trajectories across adulthood in a regional cohort and not in the same individuals [24,25]. A recent study on the Aberdeen birth cohort found that lower social class at birth was associated with higher odds of multimorbidity in middle age, which was partially mediated by educational attainment [22]. However, this study examined associations with multimorbidity at only one time point in middle age (50 to 56 years). Importantly, no previous study has examined socioeconomic patterning of multimorbidity trajectories over the lifecourse taking into account the independent effects of childhood and adulthood socioeconomic circumstances. Previous studies, for example, using electronic health records in Finland, have also reported that inequalities increase with age [10,21]. However, these may be misleading as ascertainment of chronic conditions, most of which are asymptomatic, in some cases for several years, may be biased towards either those with preexisting morbidity or healthier participants who seek healthcare. In capturing data on conditions from multiple sources, including self-report and standardised biomarker assessment on the whole cohort at multiple time points for the same individual, we overcome some concerns on biased ascertainment.

In light of secular trends in health and life expectancy, and in widening childhood and adulthood socioeconomic inequalities, these findings have significant implications for more recent cohorts who face much higher levels of multimorbidity as they age [2]. The consequence of greater prevalence of earlier life obesity and mental ill-health and their sequelae [37], but also enhanced detection and lower thresholds for medication for conditions such as diabetes and hypertension and increased survival from life threatening conditions such as myocardial infarction and some cancers, all of which potentially result in more years lived with multimorbidity.

### Strengths and limitations

This study utilises data on individuals followed up from ages 36 to 69 years enabling us to investigate the development of and increasing inequalities in multimorbidity trajectories using robust longitudinal modelling. Additionally, this study uses both periodical cross-sectional and longitudinal analyses to investigate socioeconomic patterning in multimorbidity. The NSHD was a nationally representative sample of all births in 1 week in the UK, with a >95% response rate. As with most longitudinal studies, the NSHD suffers from attrition. Nonetheless, this study included 70% of the original birth cohort (*N =* 5,362) and is reasonably representative of the British born population of this generation (and with a socioeconomic distribution and mortality rate similar to that observed nationally for this generation) [38,39]. Missing data were addressed using multiple imputation, which is a routinely used robust method. While imputation relies on the MAR assumption, which is not empirically verifiable, we increased the plausibility of the MAR assumption by including socioeconomic variables (which are known predict nonresponse or missingness) in the imputation model. This should help predict missing data with greater precision and minimising nonrandom variation in the values [40]. The NSHD is composed of only members of white European heritage, and findings may not be generalizable to the non-white British population of the same generation. Some conditions like coronary heart disease, stroke, and kidney disorders were defined by only self-report of a doctor’s diagnosis. However, this should not affect estimation of multimorbidity, as self-reporting of doctor’s diagnoses was not found to bias findings in other studies [41,42], and in NSHD, >90% agreement for diagnosis of diabetes when comparing self-report with primary care diagnosis was shown [43]. The population-based nature of the study resulted in a comprehensive set of 18 conditions over time; however, studies based on electronic health records usually consist of a larger number of conditions [10,21]. The inclusion of a limited range of common conditions and excluding more rare diseases in mid-adulthood is likely to have led to some underestimation of the extent of multimorbidity. Information on certain conditions (for instance, stroke, diabetes, and arthritis) that mostly occur in older ages was not collected in early adulthood ages, for instance, at age 36 years. However, it is possible that a small number of participants could have experienced early onset of these conditions. The definitions and criteria for diagnoses of various diseases have changed over time, making it difficult to study some conditions in greater detail, for example, all respiratory conditions had to be grouped together due to changing measurement methods and definitions over the last few decades. While there are several definitions of multimorbidity, we chose to use disease counts, which is the most commonly used definition of multimorbidity enabling comparison to other studies, and appropriate as multimorbidity is the outcome of interest (more complex definitions like weighted measures and indices are better suited to studying multimorbidity in relation to outcomes like mortality and healthcare usage) [44]. There are other important dimensions of SEP shown to be related to multimorbidity in older ages such as income and health literacy, which we were unable to take into consideration [24,45]. Lastly, we cannot exclude the possibility of residual confounding by other factors (for instance, family history of disease), which we were unable to control for, which limits causal inferences we can draw about lifetime SEP and multimorbidity trajectories.

### Clinical and policy implications

The increase in multimorbidity in older ages is both a natural consequence of longer life expectancies and significant change in lifestyle seen in recent decades. By age 53 years, 52% of our study population experienced multimorbidity, and we observe substantial inequalities in multimorbidity development. The largest increases in conditions over time were observed for those part of the cardiometabolic syndrome (obesity, hypertension, dyslipidaemia, and diabetes), and these conditions were more strongly interconnected over time as demonstrated by the network analysis. Primary care physicians are the main point of contact and ongoing care for patients with multimorbidity, with additional input from geriatricians with expertise in dealing with multimorbidity for older patients, and organ-specific clinical specialists for patients with specific conditions on a single disease basis. While there are limited clinical guidelines for addressing multimorbidity in the UK, these as yet do not include any guidance on targeted care for disadvantaged individuals at higher risk for greater multimorbidity as is clearly shown in this study [46].

The independent and long-lasting shadow of childhood SEP on both the experience and the rate of accumulation of multimorbidities in later life highlights the need for interventions early in life to minimise inequalities in later life health. Importantly, the ongoing and additional inequities observed in relation to adulthood socioeconomic factors stress the need for intervention and prevention efforts starting in childhood, but also their continued provision through the lifecourse to reduce these age-widening inequities in health and their corresponding consequences for healthy ageing, years lived with disability, and reduced life expectancy across all of society.

By 2035, the proportion of the UK population living with 4 or more conditions is expected to increase to 17% from 9.8% in 2015 [47]. Hypertension, chronic pain, obesity, depression, and anxiety are expected to be the leading contributors to multimorbidity, indicating the heterogeneity of diseases likely to co-occur and the strong interlinking between mental and physical health conditions [3]. Given that conditions like obesity, depression, and diabetes are socioeconomically patterned and more prevalent in younger generations, and combined with the projected increase in older age groups due to increasing life expectancies (those >75 years is expected to double to 13% over the next 20 years), the issue of multimorbidity, and its inequalities, will be a heavy burden that poses serious challenges for healthcare systems. Multimorbidity is linked to increased healthcare utilisation, which is largely attributable to the number of interventions required to deal with multiple chronic health conditions in individuals [48]. In addition to placing a substantial economic burden on the healthcare system, it will also require significantly more healthcare professionals and potential restructuring of healthcare provision in the near future to effectively address the issue.

The synergistic clustering of diseases at both the population and individual level is one of the mechanisms by which health inequalities develop and perpetuate in sections of the population [15,49,50]. Hence, any model developed to tackle multimorbidity and to reduce inequalities associated with socioeconomic circumstances should incorporate societal and individual-level factors. Additionally, public health policies that aim to reduce multimorbidity need to start earlier in the lifecourse, must be universal but proportional with increased targeting towards the more socioeconomically disadvantaged population. Individuals with multimorbidity living in deprived areas receive poorer healthcare (for example, shorter consultation times and lower perceived GP empathy) compared to their counterparts in affluent areas [51]. This reflects the inverse care law, which states that the availability of good medical care varies inversely with the need for it in the population served [52]. Hence, there also needs to be a focus on improving healthcare access and services at the ecological level as there is a greater concentration of individuals with multimorbidity among the socioeconomically disadvantaged.

### Final summary

In conclusion, this study shows marked and independent effects of childhood and adulthood socioeconomic circumstances on onset, rate of accumulation, and burden of multimorbidity. These inequalities widen with time and the effects of socioeconomic disadvantage in childhood on multimorbidity emerges in late adulthood and early old age. Strikingly, the most socioeconomically disadvantaged by each measure of SEP experienced an additional 0.39 conditions (childhood social class), 0.83 (adult social class), and 1.08 conditions (adult education) at age 69 years. Multimorbidity is perhaps inevitable in ageing societies with increasing life expectancies, but the inequalities as demonstrated by our study are stark. This calls not only for better access and delivery of healthcare for the more vulnerable, but also for population-based interventions in early life and through the lifecourse that reduce the impact of childhood inequalities if we are truly to address the societal impact of multimorbidity.

## Supporting information

S1 STROBE checklist STROBE Statement. (PDF)

S1 File Table A. The 18 conditions contributing to the multimorbidity score, with data sources and definitions. Table B. Example of a Delphi form circulated among 10 clinicians for consulting on how to treat the chronicity of each condition. Table C. Prevalence (%) of conditions and multimorbidity at each of the 5 age sweeps in participants from the 1946 MRC NSHD (nonimputed data). Table D. Descriptive characteristics of 3,723 participants from the 1946 MRC NSHD by socioeconomic indicators of interest. Table E. Prevalence of health conditions across the 5 age sweeps by sex in 3,723 participants from the 1946 MRC NSHD. Table F. Mean number of morbidities (health conditions) by covariates of interest in 3,723 participants from the 1946 MRC NSHD. Table G. Multimorbidity trajectories between adulthood (age 36) and old age (age 69) predicted by linear spline mixed-effects modelling in 3,723 participants from the 1946 MRC NSHD. Table H. Results from linear regression modelling assessing associations between SEP and multimorbidity in 3,723 participants from the 1946 MRC NSHD. Table I. Multimorbidity trajectories and rates in change between adulthood (age 36) and old age (age 69) predicted by linear spline mixed-effects modelling in 3,723 participants from the 1946 MRC NSHD—Models without interactions. Fig A. Results from linear regression modelling assessing associations between SEP and multimorbidity in 3,723 participants from the 1946 MRC NSHD. Fig B. Population-based predicted trajectory for multimorbidity from linear spline mixed-effects modelling in 3,723 participants from the 1946 MRC NSHD. Fig C. Population-based predicted trajectories for multimorbidity by sex from linear spline mixed-effects modelling in 3,723 participants from the 1946 MRC NSHD. NSHD, National Survey of Health and Development; SEP, socioeconomic position.
(DOCX)

## Abbreviations

MAR: missing at random
NSHD: National Survey of Health and Development
SEP: socioeconomic position
