# Supplementary material for: Socioeconomic inequalities in prevalence and development of multimorbidity across adulthood: A longitudinal analysis of the MRC 1946 National Survey of Health and Development in the UK
Source: PLoS Med. 2021 Sep 14;18(9):e1003775. doi: 10.1371/journal.pmed.1003775 (PMC8601600; doi:10.1371/journal.pmed.1003775)
Supplement: S1 STROBE checklist — (PDF) [file pmed.1003775.s001.pdf]

STROBE Statement—Checklist of items that should be included in reports of ***cohort studies***

	Item No	Recommendation
**Title and abstract**	1	(*a*) Indicate the study's design with a commonly used term in the title or the abstract<br>This is indicated in the title and abstract.
		(*b*) Provide in the abstract an informative and balanced summary of what was done and what was found –<br>The abstract includes the aims, study design, methods, main results, limitations and conclusions.
**Introduction**		
Background/rationale	2	Explain the scientific background and rationale for the investigation being reported –<br>This is summarised with sufficient detail in the Introduction.
Objectives	3	State specific objectives, including any prespecified hypotheses.<br>Last paragraph in introduction states the objectives.
**Methods**		
Study design	4	Present key elements of study design early in the paper<br>First 2 paragraphs (study participants & design) in the Methods section describe the cohort and the study design in detail.
Setting	5	Describe the setting, locations, and relevant dates, including periods of recruitment, exposure, follow-up, and data collection.<br>Described in detail in beginning of the Methods section.
Participants	6	(*a*) Give the eligibility criteria, and the sources and methods of selection of participants. Describe methods of follow-up<br>We describe in detail the study participants eligible for this study. As this study is based on the National Survey of Health and Development (NSHD), a cohort followed-up over several decades, we describe the cohort in general and the particular waves and data used for this study.
Variables	7	Clearly define all outcomes, exposures, predictors, potential confounders, and effect modifiers. Give diagnostic criteria, if applicable<br>We describe in detail all variables used in estimating multimorbidity (the outcome of interest) in three sub-sections; conditions, chronicity and multimorbidity.<br>We describe covariates in detail, including a separate section on socioeconomic variables which are the exposures of interest.
Data sources/ measurement	8*	For each variable of interest, give sources of data and details of methods of assessment (measurement). Describe comparability of assessment methods if there is more than one group<br>Described in detail (as mentioned above). Additionally, Table 1, and Supplemental Tables 1 and 2 also provide information on the different variables (the 18 conditions used in estimating multimorbidity) and sources of data.
Bias	9	Describe any efforts to address potential sources of bias<br>In longitudinal studies one of the biggest issues is attrition over time. We report on rates of missing data and conduct imputation to account for attrition over time.
Study size	10	Explain how the study size was arrived at<br>This is described in the last 2 paragraphs of the 'study participants and design' section in the beginning of the Methods.
Quantitative variables	11	Explain how quantitative variables were handled in the analyses. If applicable, describe which groupings were chosen and why

		We describe in detail all variables used to estimate multimorbidity, our outcome of interest. We describe how we estimated multimorbidity, and how we categorised covariates of interests (socioeconomic variables and gender).
Statistical methods	12	(*a*) Describe all statistical methods, including those used to control for confounding<br>This is described in the Methods-Analysis section. We separately describe the different statistical analyses used: summary statistics, cross-sectional and longitudinal analyses.
		(*b*) Describe any methods used to examine subgroups and interactions<br>We describe in detail how we estimated the development of multimorbidity over time in the full study sample and then separately by the main covariates of interest (three socioeconomic variables: childhood and adulthood social class and educational level). The only interactions we tested were those between socioeconomic and time (spline variables) in the longitudinal analysis. This is described in detail in the second last paragraph in the statistical analysis section.
		(*c*) Explain how missing data were addressed<br>We addressed missing data using multiple imputation. This is described at the end of the statistical analysis section.
		(*d*) If applicable, explain how loss to follow-up was addressed<br>We describe missing data and numbers at each follow-up. The study was restricted to those participants that attended any of the ages 36, 43, 53, 63 and 69 waves and missing data was addressed with the use of multiple imputation. Frequencies and distributions of non-imputed and imputed variables were largely similar for most conditions and are presented in Supplementary Table 3 and Table 1 respectively.
		(*e*) Describe any sensitivity analyses<br>Descriptive analysis with and without multiple imputation.
**Results**		
Participants	13*	(a) Report numbers of individuals at each stage of study—eg numbers potentially eligible, examined for eligibility, confirmed eligible, included in the study, completing follow-up, and analysed<br>We described attrition (number of participants) lost during follow-up and the final number of participants included in this study in the Methods section.
		(b) Give reasons for non-participation at each stage<br>The reasons for non-participation vary and sometimes are not known. This information is briefly presented in the manuscript, where known, in the participants section under the subheading ‘attrition’.
Descriptive data	14*	(a) Give characteristics of study participants (eg demographic, clinical, social) and information on exposures and potential confounders<br>The first paragraph in the Results section summarises the main characteristics of the study participants. We also describe how multimorbidity develops over time and differences in multimorbidity by socioeconomic covariates of interest.
		(b) Indicate number of participants with missing data for each variable of interest
		This information is presented in Suppl Table 3
Outcome data	15*	Report numbers of outcome events or summary measures over time<br>Multimorbidity – the outcome of interest – is described in detail including how it develops over time (at the five different ages between ages 36 and 69 years) in Table 1.
Main results	16	(*a*) Give unadjusted estimates and, if applicable, confounder-adjusted estimates and their precision (eg, 95% confidence interval). Make clear which confounders were

		adjusted for and why they were included<br>We provide the unadjusted values of multimorbidity at each of the five ages over follow-up, as well as unadjusted estimates of multimorbidity by socioeconomic covariates of interest. We also provide the adjusted estimates for multimorbidity (for example, estimates for multimorbidity mutually adjusted for all three socioeconomic variables and gender). This is done for both cross-sectional (adjusted estimates from multivariable linear regression modelling) and longitudinal (adjusted estimates from mixed-effects models) analyses. All regression estimates are reported with corresponding 95% Cis in both text and tables.
		(*b*) Report category boundaries when continuous variables were categorized<br>NA, no continuous variables were categorised.
		(*c*) If relevant, consider translating estimates of relative risk into absolute risk for a meaningful time period<br>NA, outcome is a continuous variable.
Other analyses	17	Report other analyses done—eg analyses of subgroups and interactions, and sensitivity analyses<br>We describe results of interactions (between socioeconomic variables and spline variables) included in mixed-effects models used for longitudinal analysis, how these can be interpreted as well as graphs to visually display the interactions (for example how multimorbidity trajectories vary by socioeconomic variables) over time.
**Discussion**		
Key results	18	Summarise key results with reference to study objectives<br>This is described in detail in the first paragraph of the Discussion.
Limitations	19	Discuss limitations of the study, taking into account sources of potential bias or imprecision. Discuss both direction and magnitude of any potential bias.<br>We describe the limitations of the study including potential limitations in our estimation of multimorbidity in the discussion, paras 2,4,5.
Interpretation	20	Give a cautious overall interpretation of results considering objectives, limitations, multiplicity of analyses, results from similar studies, and other relevant evidence<br>See Discussion all paragraphs.
Generalisability	21	Discuss the generalisability (external validity) of the study results.<br>We discuss that generalisability of results could be limited (for example with non-White individuals).
**Other information**		
Funding	22	Give the source of funding and the role of the funders for the present study and, if applicable, for the original study on which the present article is based.<br>Source of funding and all details have been provided in the online system as requested by the journal

*Give information separately for exposed and unexposed groups.

Note: An Explanation and Elaboration article discusses each checklist item and gives methodological background and published examples of transparent reporting. The STROBE checklist is best used in conjunction with this article (freely available on the Web sites of PLoS Medicine at http://www.plosmedicine.org/, Annals of Internal Medicine at http://www.annals.org/, and Epidemiology at http://www.epidem.com/). Information on the STROBE Initiative is available at http://www.strobe-statement.org.
