# Supplementary material for: Socioeconomic inequalities in prevalence and development of multimorbidity across adulthood: A longitudinal analysis of the MRC 1946 National Survey of Health and Development in the UK
Source: PLoS Med. 2021 Sep 14;18(9):e1003775. doi: 10.1371/journal.pmed.1003775 (PMC8601600; doi:10.1371/journal.pmed.1003775)
Supplement: S1 File — Table A. The 18 conditions contributing to the multimorbidity score, with data sources and definitions. Table B. Example of a Delphi form circulated among 10 clinicians for consulting on how to treat the chronicity of each condition. Table C. Prevalence (%) of conditions and multimorbidity at each of the 5 age sweeps in participants from the 1946 MRC NSHD (nonimputed data). Table D. Descriptive characteristics of 3,723 participants from the 1946 MRC NSHD by socioeconomic indicators of interest. Table E. Prevalence of health conditions across the 5 age sweeps by sex in 3,723 participants from the 1946 MRC NSHD. Table F. Mean number of morbidities (health conditions) by covariates of interest in 3,723 participants from the 1946 MRC NSHD. Table G. Multimorbidity trajectories between adulthood (age 36) and old age (age 69) predicted by linear spline mixed-effects modelling in 3,723 participants from the 1946 MRC NSHD. Table H. Results from linear regression modelling assessing associations between SEP and multimorbidity in 3,723 participants from the 1946 MRC NSHD. Table I. Multimorbidity trajectories and rates in change between adulthood (age 36) and old age (age 69) predicted by linear spline mixed-effects modelling in 3,723 participants from the 1946 MRC NSHD—Models without interactions. Fig A. Results from linear regression modelling assessing associations between SEP and multimorbidity in 3,723 participants from the 1946 MRC NSHD. Fig B. Population-based predicted trajectory for multimorbidity from linear spline mixed-effects modelling in 3,723 participants from the 1946 MRC NSHD. Fig C. Population-based predicted trajectories for multimorbidity by sex from linear spline mixed-effects modelling in 3,723 participants from the 1946 MRC NSHD. NSHD, National Survey of Health and Development; SEP, socioeconomic position. (DOCX) [file pmed.1003775.s002.docx]

**Supplementary file**

**Socioeconomic inequalities in prevalence and development of multimorbidity across adulthood: A longitudinal analysis of the MRC 1946 National Survey of Health & Development in the UK**

Amal R. Khanolkar^1,2^, Nishi Chaturvedi^1^, Valerie Kuan Po Ai^3^ Daniel Davis^1^, Alun Hughes^1^, Marcus Richards^1^, David Bann^4^, Praveetha Patalay^1,4^

1. MRC Unit for Lifelong Health and Ageing at UCL, London

2. Department of Global Public Health, Karolinska Institutet, Stockholm,

3. Institute of Health Informatics, UCL, London

4. Centre for Longitudinal Studies, UCL, London

**Supplemental Table A. The eighteen conditions contributing to the multimorbidity score, with data sources and definitions**

| **Conditions** | **Source of information** | **Definitions, criteria/cut-offs** | **Ages at which data available** | **Carried forward or considered chronic** |
| --- | --- | --- | --- | --- |
| **Obesity** | Measured height and weight | BMI≥30 kg/m^2^ | All | No |
| **Hypertension** | Blood pressure values & medication | Current SBP≥160 mmHg or current DBP≥100 mmHg | All | Yes |
| **Dyslipidaemia** | Cholesterol/HDL values & medications | Total:HDL cholesterol ratio ≥6.0mmol/l | 53, 63 & 69 | Yes |
| **Diabetes** | Self-report & medication |  | 43, 53, 63 & 69 | Yes |
| **CHD** | Self-report |  | 43, 53, 63 & 69 | Yes |
| **Stroke** | Self-report |  | 53, 63 & 69 | Yes |
| **Osteoarthritis** | Self-report & medication |  | 63 & 69 | Yes |
| **Rheumatoid Arthritis** | Self-report & medication |  | 53, 63 & 69 | Yes |
| **Anaemia** | Haemoglobin values & medication | Haemoglobin <13 g/dl (males) or haemoglobin <12 g/dl (females) | 53, 63 & 69 | No |
| **Skin disorders** | Self-report & Medication |  | 36, 43, 53 & 69 | No |
| **Respiratory disorders** | Self-report & medication |  | All | No |
| **Gastrointestinal disorders** | Medication |  | All | No |
| **Kidney disorders** | Self-report |  | 36, 43, 53 & 69 | No |
| **Parkinson disease** | Self-report & medication |  | 63 & 69 | Yes |
| **Cancer** | National statistics (linked cancer registry data) |  | All | Yes |
| **Epilepsy** | Medication |  | All | No |
| **Depression** | Self-report & medication |  | All | No |
| **Psychotic disorders** | Medication |  | All | Yes |

**Supplemental Table B. Example of a Delphi form circulated among ten clinicians for consulting on how to treat the chronicity of each condition**

| **Condition** | **Source variables** | **Condition carried forward?**  **Yes/No** | **Reasoning for the decision** |
| --- | --- | --- | --- |
| Diabetes | Self-report, Medication |  |  |
| Hypertension | BP values, medication |  |  |
| Dyslipidemia | Cholesterol/HDL values, medications |  |  |
| Obesity | BMI values only |  |  |
| CHD | Self-report only |  |  |
| Stroke | Self-report only |  |  |
|  |  |  |  |
| Anaemia | Haemoglobin values, medication |  |  |
| Rheumatoid Arthritis | Self-report, medication |  |  |
| Osteo Arthritis | Self-report only |  |  |
| Cancer | Linked cancer registry data |  |  |
| Kidney disease | Self-report only |  |  |
| Parkinson’s | Self-report, medication |  |  |
| Respiratory illness (including asthma, bronchitis) | Self-report, medication |  |  |
| Skin conditions (e.g. eczema) | Self-report,  medication |  |  |
| Epilepsy | Medication only |  |  |
| Psychotic disorders | Medication only |  |  |
| Depression | Self-report, medication |  |  |

**Supplemental Table C. Prevalence (%) of conditions and multimorbidity at each of the five age sweeps in participants from the 1946 MRC National Survey of Health and Development (non-imputed data)**

| **Condition** | **Age (prevalence %, N)** | | | | |
| --- | --- | --- | --- | --- | --- |
|  | **Age 36** | **Age 43** | **Age 53** | **Age 63** | **Age 69** |
| **Obesity (%)**  **N** | 6.6  3,064 | 12.3  3,225 | 24.2  2,946 | 29.2  2,218 | 30.3  2,111 |
| **Hypertension (%)**  **N** | 6.2  3,288 | 12.1  3,587 | 27.9  3,670 | 36.7  3,716 | 40.1  3,716 |
| **Dyslipidaemia (%)**  **N** | N.A. | N.A. | 8.3  2,988 | 25.3  3,191 | 35.7  3,211 |
| **Diabetes (%)**  **N** | N.A. | 1.1  3,254 | 2.6  3,673 | 6.7  3,718 | 10.1  3,718 |
| **CHD (%)**  **N** | N.A. | 3.3  3,262 | 7.8  3,443 | 13.9  3,517 | 14.8  3,522 |
| **Stroke (%)**  **N** | N.A. | N.A. | 0.8  2,988 | 1.9  3,166 | 3.9  3,242 |
| **Osteoarthritis (%)**  **N** | N.A. | N.A. | N.A. | 7.7  2,406 | 20.5  2,723 |
| **Rheumatoid Arthritis (%)**  **N** | N.A. | N.A. | 0.3  2,988 | 1.3  3,191 | 3.6  3,215 |
| **Anaemia (%)**  **N** | N.A. | N.A. | 8.2  2,988 | 5.1  2,657 | 8.7  2,134 |
| **Skin disorders (%)**  **N** | 10.5  3,322 | 18.5  3,262 | 18.8  2,988 | N.A. | 14.2  2,131 |
| **Respiratory disorders (%)**  **N** | 15.7  3,322 | 28.3  3,262 | 16.7  2,988 | 18.8  2,658 | 23.4  2,574 |
| **Gastrointestinal disorders(%)**  **N** | 1.9  3,322 | 8.4  3,262 | 6.6  2,988 | 16.9  2,656 | 24.5  2,112 |
| **Kidney disorders**  **N** | 4.8  3,322 | 13.6  3,262 | 12.5  2,958 | N.A. | 2.3  2,194 |
| **Parkinson’s disease (%)**  **N** | N.A. | N.A. | N.A. | 0.4  2,656 | 0.8  2,795 |
| **Cancer (%)**  **N** | 0.6  3,723 | 1.7  3,723 | 5.6  3,723 | 14.2  3,723 | 19.6  3,723 |
| **Epilepsy (%)**  **N** | 1.1  3,322 | 1.5  3,262 | 1.9  2,988 | 1.6  3,166 | 3.3  2,194 |
| **Depression (%)**  **N** | 17.9  3,322 | 15.6  3,262 | 21.7  2,989 | 20.5  2,659 | 19.7  2,137 |
| **Psychotic disorders (%)**  **N** | 0.6  3,322 | 1.1  3,594 | 1.4  3,674 | 1.8  3,719 | 1.9  3,719 |
| **Number of conditions (mean)** | 0.6 | 1.1 | 1.4 | 1.6 | 2.1 |

**N.A.: Not Applicable – indicates data for that condition was not collected at the particular age**

**Supplemental Table D. Descriptive characteristics of 3,723 participants from the 1946 MRC National Survey of Health and Development by socioeconomic indicators of interest**

All figures are percentages (95% CI)

|  | **All**  **N=3,723** | **Men**  **N=1,880** | **Women**  **N=1,843** |
| --- | --- | --- | --- |
|  |  |  |  |
| **Childhood Social class** |  |  |  |
| Professional/intermediate | 25.9 (24.4-27.3) | 26.5 (24.5-28.6) | 25.2 (23.1-27.2) |
| Non-manual | 16.1 (14.9-17.3) | 15.9 (14.2-17.6) | 16.3 (14.5-18) |
| Manual | 32.3 (30.1-33.8) | 32.2 (30-34.3) | 32.4 (30.2-34.6) |
| Partly/unskilled | 25.7 (24.3-27.2) | 25.4 (23.4-27.4) | 26.1 (24-28.2) |
|  |  |  |  |
| **Adulthood Social Class** |  |  |  |
| Professional/intermediate | 41.8 (40.2-43.4) | 50.2 (47.9-52.5) | 33.2 (31-35.4) |
| Non-manual | 23.4 (21.9-27.8) | 9.1 (7.8-10.4) | 38 (35.7-40.2) |
| Manual | 18.5 (17.3-19.8) | 29.3 (27.2-31.4) | 7.4 (6.3-8.7) |
| Partly/unskilled | 16.3 (15.1-17.5) | 11.3 (9.9-12.8) | 21.4 (19.5-23.3) |
|  |  |  |  |
| **Educational Attainment** |  |  |  |
| University degree | 34.5 (32.9-26.1) | 31.5 (29.4-33.7) | 37.4 (35.2-39.7) |
| None | 53.6 (51.9-55.3) | 52.3 (49.9-54.6) | 55 (52.6-57.4) |
| GCE/School leaving certificate | 11.9 (10.8-12.9) | 16.2 (14.4-17.8) | 7.5 (6.3-8.8) |

**Supplemental Table E. Prevalence of health conditions across the five age sweeps by sex in 3,723 participants from the 1946 MRC National Survey of Health and Development**

All figures are percentages (95% CI)

| **Condition** | **Age sweep**  **Males, N=1,880 & Females, N=1,843** | | | | | | | | | |
| --- | --- | --- | --- | --- | --- | --- | --- | --- | --- | --- |
|  | **Age 36** | | **Age 43** | | **Age 53** | | **Age 63** | | **Age 69** | |
|  | Males | Females | Males | Females | Males | Females | Males | Females | Males | Females |
| **Obesity** | 6.7  (5.4-7.8) | 7.2  (5.8-8.4) | 11.4 (9.8-13) | 14.1  (12.5-15.8) | 23.6  (21.4-25.8) | 26.2  (23.9-28.5) | 31.4  (28.9-33.9) | 33  (29.8-36) | 34.3  (31.2-37.5) | 34.4  (31.6-37.2) |
| **Hypertension** | 8.3  (6.9-9.6) | 4.2  (3.2-5.2) | 16.6  (14.7-18.4) | 10  (8.4-11.5) | 38.8  (36.3-41.4) | 25.8  (23.6-27.9) | 50.8  (48.2-53.6) | 38.5  (36-41) | 58.1  (55.4-60.8) | 45.6  (42.8-48.4) |
| **Dyslipidaemia** | - | - | - | - | 12.4  (10-6-14.1) | 5.7  (4.5-7.1) | 36.9  (34-39.7) | 27.4  (24.8-29.8) | 54  (50.9-56.9) | 41.5  (38.7-44.4) |
| **Diabetes** | - | - | 1.8  (1.1-2.5) | 1.2  (0.5-1.7) | 3.6  (2.6-4.5) | 2.7  (1.9-3.5) | 12.1  (10.2-13.9) | 9.3  (7.6-10.9) | 18.1  (15.9-20.3) | 14.4  (12.4-16.3) |
| **CHD** | - | - | 3.9  (2.9-4.9) | 3.7  (2.6-4.7) | 12.3  (10.5-14) | 10.9  (9-12.7) | 22.3  (20-24.5) | 18.2  (15.9-20.4) | 25  (22.6-27.5) | 20  (18.2-22.6) |
| **Stroke** | - | - | - | - | 2.7  (1.4-3.9) | 2.3  (0.9-3.6) | 7.1  (5.1-9) | 5.7  (3.7-7.7) | 11.6  (9.6-13.6) | 9  (6.9-10.9) |
| **Osteoarthritis** | - | - | - | - | - | - | 8  (5.8-9.9) | 10.8  (9-12.7) | 23.8  (20.8-26.8) | 31.2  (28.3-34.2) |
| **Rheumatoid Arthritis** | - | - | - | - | 1.3  (1-2.4) | 1.4  (0.4-2.2) | 3.9  (1.9-5.9) | 4.5  (2.7-6.2) | 9.5  (6.4-12.5) | 10  (7.5-12.4) |
| **Anaemia** | - | - | - | - | 4  (2.6-5.2) | 14.2  (12.4-16) | 6.4  (4.4-8.3) | 7.3  (5.4-9.2) | 12.2  (9.7-14.7) | 12.8  (10.3-15.3) |
| **Skin disorders** | 11  (9.4-12.5) | 10.6  (9-12) | 19  (16.9-20.9) | 18.4  (16.5-20.2) | 19.5  (17.4-21.5) | 18.7  (16.7-20.6) | - | - | 15.8  (13.4-18.1) | 16.4  (14-18.7) |
| **Respiratory disorders** | 16.3  (14.5-18.1) | 15.8  (14.1-17.6) | 27.3  (25.2-29.4) | 29.6  (27.4-31.8) | 16.4  (14.3-18.4) | 18.6  (16.5-20.6) | 18.3  (15.9-20.5) | 22.6  (20.3-24.8) | 24.2  (21.6-26.7) | 27.1  (24.5-29.7) |
| **Gastrointestinal disorders** | 2.6  (1.7-3.5) | 1.8  (0.1-0.3) | 8.6  (7.2-9.9). | 9.2  (7.7-10.6) | 7.8  (6.3-9.4) | 8  (6.5-9.5) | 19.3  (16.7-21.9) | 22.1  (19.6-24.5) | 26.5  (22.8-30.1) | 30.1  (26.5-33.7) |
| **Kidney disorders** | 1.8  (1.1-2.5) | 8  (6.7-9.3) | 7.7  (6.4-9) | 20  (15-19.3) | 9.5  (7.8-11.2) | 17.2  (15-19.3) | - | - | 5  (2.4-7.6) | 5.1  (2.8-7.3) |
| **Parkinson’s disease** | - | - | - | - | - | - | 2.8  (1.1-4.5) | 2.4  (0.9-3.9) | 5.9  (4.1-7.8) | 5  (2.9-7) |
| **Cancer** | 0.5  (0.1-0.7) | 0.7  (0.3-1.9) | 1  (0.7-1.6) | 2.3  (1.6-2.9) | 3.5  (2.6-4.3) | 7.7  (6.4-8.9) | 12  (10.5-13.4) | 16.4  (14.7-18.1) | 18.5  (16.7-20.2) | 20.7  (18.9-22.5) |
| **Epilepsy** | 1.6  (0.1-2.3) | 1.3  (0.7-1.9) | 2  (1.1-2.8) | 2.2  (1.3-3.1) | 4.5  (2.9-6) | 3.9  (2.4-5.3) | 3.4  (2.1-4.7) | 2.8  (1.7-3.7) | 8.4  (6.5-10.4) | 8.2  (6-10.4) |
| **Depression** | 13.3  (11.6-15.1) | 23.5  (21.4-25.5) | 13.2  (11.4-14.9) | 19.8  (17.9-21.7) | 18.4  (16.2-20.5) | 27.8  (25.5-30.1) | 18.5  (16.2-20.8) | 28.4  (26-30.7) | 20.5  (18.2-22.9) | 28.6  (25.9-31.2) |
| **Psychotic disorders** | 1.1  (0.4-1.8) | 0.5  (0.3-1.3) | 2.3  (1.4-3.2) | 1.8  (1-2.6) | 4.3  (2.8-3.2) | 3.5  (2-4.9) | 6.6  (4.6-8.6) | 5.4  (3.7-7.1) | 8.6  (6.3-10.9) | 7.5  (5.6-9.3) |
| **Multimorbidity**  **(mean)** | 0.63  (0.59-0.67) | 0.74  (0.69-0.780 | 1.15  (1.09-1.20) | 1.32  (1.26-1.38) | 1.82  (1.74-1.91) | 1.95  (1.86-2.03) | 2.59  (2.48-2.71) | 2.55  (2.43-2.66) | 3.80  (3.64-3.96) | 3.68  (3.52-3.84) |

**Supplemental Table F. Mean number of morbidities (health conditions) by covariates of interest in 3,723 participants from the 1946 MRC National Survey of Health and Development**

|  | **Mean number of morbidities (SD)** | | | | |
| --- | --- | --- | --- | --- | --- |
|  | **Age 36** | **Age 43** | **Age 53** | **Age 63** | **Age 69** |
| **All Participants** | 0.68 (0.86) | 1.22 (1.18) | 1.86 (1.57) | 2.53 (2.04) | 3.68 (2.62) |
| **Sex** |  |  |  |  |  |
| Men | 0.62 (0.82) | 1.14 (1.12) | 1.80 (1.52) | 2.55 (2.03) | 3.73 (2.58) |
| Women | 0.73 (0.89) | 1.31 (1.24) | 1.92 (1.62) | 2.51 (2.05) | 3.62 (2.66) |
|  |  |  |  |  |  |
| **Childhood social class** |  |  |  |  |  |
| Professional/intermediate | 0.62 (0.83) | 1.13 (1.11) | 1.72 (1.47) | 2.18 (1.88) | 3.15 (2.38) |
| Non-manual | 0.65 (0.81) | 1.22 (1.17) | 1.74 (1.43) | 2.32 (1.82) | 3.39 (2.33) |
| Manual | 0.71 (0.87) | 1.24 (1.23) | 1.95 (1.64) | 2.73 (2.12) | 3.92 (2.72) |
| Partly/unskilled | 0.72 (0.90) | 1.30 (1.21) | 1.97 (1.64) | 2.78 (2.15) | 4.09 (2.76) |
|  |  |  |  |  |  |
| **Adulthood social class** |  |  |  |  |  |
| Professional/intermediate | 0.60 (0.80) | 1.14 (1.10) | 1.67 (1.43) | 2.25 (1.86) | 3.24 (2.35) |
| Non-manual | 0.70 (0.86) | 1.25 (1.22) | 1.87 (1.57) | 2.41 (1.99) | 3.51 (2.57) |
| Manual | 0.67 (0.85) | 1.20 (1.15) | 1.92 (1.53) | 2.80 (2.08) | 4.08 (2.68) |
| Partly/unskilled | 0.85 (0.99) | 1.44 (1.34) | 2.28 (1.85) | 3.14 (2.30) | 4.59 (3.68) |
|  |  |  |  |  |  |
| **Educational level** |  |  |  |  |  |
| University degree | 0.59 (0.77) | 1.13 (1.09) | 1.62 (1.36) | 2.07 (1.75) | 2.87 (2.19) |
| GCE/school leaving certificate | 0.64 (0.83) | 1.15 (1.13) | 1.74 (1.48) | 2.33 (1.92) | 3.39 (2.43) |
| None | 0.77 (0.92) | 1.37 (1.28) | 2.13 (1.72) | 3.02 (2.20) | 4.41 (2.84) |

**Supplemental Table G. Multimorbidity trajectories between adulthood (age 36) and old age (age 69) predicted by linear spline mixed-effects modelling in 3,723 participants from the 1946 MRC National Survey of Health and Development**

|  | **Model 1: Multimorbidity in full sample** | **Model 2:**  **Childhood social class^a^** | **Model 3:**  **Adulthood social class^b^** | **Model 4:**  **Educational level** |
| --- | --- | --- | --- | --- |
|  | **β 95% CI** | **β 95% CI** | **β 95% CI** | **β 95% CI** |
| **Spline variables** |  |  |  |  |
| *Change in multimorbidity score* *or increase in number of conditions per year in each spline variable* | | | | |
| **Ages 36 to 43 (1982-89)** | **0.08 0.07, 0.09** | **0.08 0.07, 0.09** | **0.53* 0.45, 0.61** | **0.08 0.07, 0.09** |
| **Ages 43 to 53 (1989-99)** | **0.07 0.06, 0.07** | **0.06 0.05, 0.07** | **0.54* 0.45, 0.63** | **0.06 0.05, 0.07** |
| **Ages 53 to 63 (1999-09)** | **0.07 0.06, 0.08** | **0.46* 0.35, 0.57** | **0.59* 0.50, 0.67** | **0.07 0.06, 0.07** |
| **Ages 63 to 69 (2009-15)** | **0.19 0.18, 0.20** | **1.00* 0.86, 1.13** | **1.00* 0.89, 1.11** | **0.19 0.18, 0.20** |
|  |  |  |  |  |
| **Childhood Social Class** |  |  |  |  |
| Professional/intermediate |  | **Ref** | **Ref** | **Ref** |
| Non-manual |  | 0.04 -0.05, 0.14 | 0.03 -0.06, 0.12 | 0.03 -0.07, 0.12 |
| Manual |  | 0.07 -0.01, 0.15 | 0.02 -0.05, 0.10 | 0.03 -0.05, 0.10 |
| Partly skilled/unskilled |  | 0.08 -0.02, 0.17 | 0.02 -0.07, 0.11 | 0.02 -0.08, 0.11 |
| **Interactions between spline variables & childhood social class** |  |  |  |  |
| Ages 53 to 63 * Non-manual |  | 0.10* -0.07, 0.28 |  |  |
| Ages 53 to 63 * Manual |  | **0.35* 0.19, 0.50** |  |  |
| Ages 53 to 63 * Partly skilled/unskilled |  | **0.37* 0.19, 0.54** |  |  |
| Ages 63 to 69 * Non-manual |  | 0.10* -0.08, 0.28 |  |  |
| Ages 63 to 69 * Manual |  | **0.20* 0.30, 0.37** |  |  |
| Ages 63 to 69 * Partly skilled/unskilled |  | **0.36* 0.14, 0.52** |  |  |
| **Adulthood Social Class** |  |  |  |  |
| Professional/intermediate |  | **Ref** | **Ref** | **Ref** |
| Non-manual |  | 0.04 -0.04, 0.13 | 0.03 -0.07, 0.13 | 0.04 -0.04, 0.13 |
| Manual |  | 0.03 -0.05, 0.12 | 0.06 -0.04, 0.17 | 0.03 -0.06, 0.12 |
| Partly skilled/unskilled |  | **0.17 0.07, 0.27** | **0.17 0.05, 0.29** | **0.17 0.07, 0.27** |
| **Interactions between spline variables & adulthood social class** |  |  |  |  |
| Ages 36 to 43 * Non-manual |  |  | 0.02* -0.10, 0.15 |  |
| Ages 36 to 43 * Manual |  |  | -0.01* -0.13, 0.13 |  |
| Ages 36 to 43 * Partly skilled/unskilled |  |  | 0.06* -0.08, 0.21 |  |
| Ages 43 to 53 * Non-manual |  |  | 0.08* -0.05, 0.22 |  |
| Ages 43 to 53 * Manual |  |  | **0.18* 0.03, 0.34** |  |
| Ages 43 to 53 * Partly skilled/unskilled |  |  | **0.31* 0.15, 0.47** |  |
| Ages 53 to 63 * Non-manual |  |  | -0.04* -0.17, 0.10 |  |
| Ages 53 to 63 * Manual |  |  | **0.31* 0.16, 0.48** |  |
| Ages 53 to 63 * Partly skilled/unskilled |  |  | **0.29* 0.10, 0.48** |  |
| Ages 63 to 69 * Non-manual |  |  | 0.11* -0.05, 0.27 |  |
| Ages 63 to 69 * Manual |  |  | **0.30* 0.12, 0.49** |  |
| Ages 63 to 69 * Partly skilled/unskilled |  |  | **0.49* 0.25, 0.73** |  |
| **Educational Attainment** |  |  |  |  |
| University degree |  | **Ref** | **Ref** | **Ref** |
| GCE/School leaving certificate |  | **-0.05 -0.15, 0.05** | -0.05 -0.15, 0.53 | -0.05 -0.15, 0.05 |
| None |  | **0.04 -0.09, 0.16** | 0.03 -0.08, 0.16 | 0.04 -0.09, 0.16 |
| **Sex** |  |  |  |  |
| Males |  | **Ref** | **Ref** | **Ref** |
| Females |  | **0.13 0.07, 0.19** | **0.13 0.07, 0.19** | **0.13 0.07, 0.19** |
| **Multimorbidity score (number of conditions) at age 36 *(constant)*** | **0.69 0.65, 0.73** | **0.40 0.28, 0.53** | **0.43 0.31, 0.56** | **0.44 0.32, 0.56** |
| ^a^Model for childhood social class: includes interactions between childhood social class and spline variables for ages 53-63 and 63-69. ^b^Model for adulthood social class: includes interactions between adulthood social class and all four spline variables.  *Estimates for variables including interactions are interpreted as change in multimorbidity score per spline period, for e.g., 0.59 indicates 0.59 more conditions between ages 53 to 63.  Note: Text in bold indicate 95% confidence intervals that do not include zero | | | | |

**Supplemental Table H. Results from linear regression modelling assessing associations between socioeconomic position and multimorbidity in 3,723 participants from the** **1946 MRC National Survey of Health and Development**

|  | **Change in multimorbidity score** | | | | | | | |
| --- | --- | --- | --- | --- | --- | --- | --- | --- |
| **Covariates** | **Model 1^a^**  **β** | **95% CI** | **Model 2^b^**  **β** | **95% CI** | **Model 3^c^**  **β** | **95% CI** | **Model 4^d^**  **β** | **95% CI** |
| **1982, Age 36** | | | | | | | | |
| **Sex** |  |  |  |  |  |  |  |  |
| Men | Ref |  | Ref |  | Ref |  | Ref |  |
| Women | **0.11** | **[0.05,0.17]** | **0.09** | **[0.02,0.15]** | **0.1** | **[0.04,0.16]** | **0.09** | **[0.02,0.15]** |
| **Childhood Social Class** |  |  |  |  |  |  |  |  |
| Professional/intermediate | Ref |  |  |  |  |  | Ref |  |
| Non-manual | 0.03 | [-0.06,0.13] |  |  |  |  | 0.03 | [-0.07,0.12] |
| Manual | **0.09** | **[0.01,0.17]** |  |  |  |  | 0.05 | [-0.04,0.13] |
| Partly skilled/unskilled | **0.1** | **[0.02,0.18]** |  |  |  |  | 0.03 | [-0.06,0.12] |
| **Adulthood Social Class** |  |  |  |  |  |  |  |  |
| Professional/intermediate |  |  | Ref |  |  |  | Ref |  |
| Non-manual |  |  | 0.06 | [-0.03,0.14] |  |  | 0.04 | [-0.04,0.13] |
| Manual |  |  | **0.09** | **[0.00,0.17]** |  |  | 0.05 | [-0.04,0.15] |
| Partly skilled/unskilled |  |  | **0.23** | **[0.14,0.32]** |  |  | **0.18** | **[0.08,0.28]** |
| **Educational Attainment** |  |  |  |  |  |  |  |  |
| University degree |  |  |  |  | Ref |  | Ref |  |
| None |  |  |  |  | **0.15** | **[0.05,0.26]** | 0.06 | [-0.07,0.18] |
| GCE/School leaving cert |  |  |  |  | 0.03 | [-0.07,0.13] | -0.01 | [-0.11,0.09] |
|  |  |  |  |  |  |  |  |  |
| **1989, Age 43** | | | | | | | | |
| **Sex** |  |  |  |  |  |  |  |  |
| Men | Ref |  | Ref |  | Ref |  | Ref |  |
| Women | **0.17** | **[0.09,0.25]** | **0.15** | **[0.06,0.24]** | **0.16** | **[0.08,0.24]** | **0.15** | **[0.06,0.24]** |
| **Childhood Social Class** |  |  |  |  |  |  |  |  |
| Professional/intermediate | Ref |  |  |  |  |  | Ref |  |
| Non-manual | 0.08 | [-0.05,0.22] |  |  |  |  | 0.08 | [-0.05,0.21] |
| Manual | **0.11** | **[0.00,0.22]** |  |  |  |  | 0.05 | [-0.07,0.16] |
| Partly skilled/unskilled | **0.17** | **[0.05,0.29]** |  |  |  |  | 0.07 | [-0.06,0.20] |
| **Adulthood Social Class** |  |  |  |  |  |  |  |  |
| Professional/intermediate |  |  | Ref |  |  |  | Ref |  |
| Non-manual |  |  | 0.05 | [-0.06,0.17] |  |  | 0.03 | [-0.09,0.15] |
| Manual |  |  | 0.1 | [-0.02,0.22] |  |  | 0.03 | [-0.10,0.16] |
| Partly skilled/unskilled |  |  | **0.28** | **[0.15,0.40]** |  |  | **0.17** | **[0.03,0.32]** |
| **Educational Attainment** |  |  |  |  |  |  |  |  |
| University degree |  |  |  |  |  |  |  |  |
| None |  |  |  |  | **0.21** | **[0.07,0.36]** | 0.13 | [-0.04,0.30] |
| GCE/School leaving cert |  |  |  |  | -0.01 | [-0.14,0.12] | -0.05 | [-0.18,0.09] |
| **1999, Age 53** | | | | | | | | |
| **Sex** |  |  |  |  |  |  |  |  |
| Men | Ref |  | Ref |  | Ref |  | Ref |  |
| Women | 0.12 | [0.01,0.23] | 0.07 | [-0.05,0.19] | 0.09 | [-0.03,0.20] | 0.06 | [-0.07,0.18] |
| **Childhood Social Class** |  |  |  |  |  |  |  |  |
| Professional/intermediate | Ref |  |  |  |  |  | Ref |  |
| Non-manual | 0.02 | [-0.16,0.20] |  |  |  |  | -0.01 | [-0.18,0.17] |
| Manual | **0.24** | **[0.09,0.38]** |  |  |  |  | 0.09 | [-0.07,0.24] |
| Partly skilled/unskilled | **0.26** | **[0.10,0.42]** |  |  |  |  | 0.04 | [-0.14,0.22] |
| **Adulthood Social Class** |  |  |  |  |  |  |  |  |
| Professional/intermediate |  |  | Ref |  |  |  | Ref |  |
| Non-manual |  |  | **0.17** | **[0.02,0.33]** |  |  | 0.12 | [-0.04,0.27] |
| Manual |  |  | **0.27** | **[0.10,0.44]** |  |  | 0.13 | [-0.05,0.31] |
| Partly/unskilled |  |  | **0.61** | **[0.43,0.78]** |  |  | **0.43** | **[0.24,0.62]** |
| **Educational Attainment** |  |  |  |  |  |  |  |  |
| University degree |  |  |  |  | Ref |  | Ref |  |
| None |  |  |  |  | **0.5** | **[0.29,0.71]** | **0.28** | **[0.04,0.53]** |
| GCE/School leaving cert |  |  |  |  | 0.1 | [-0.08,0.29] | 0.02 | [-0.18,0.22] |
| **2009, Age 63** | | | | | | | | |
| **Sex** |  |  |  |  |  |  |  |  |
| Men | Ref |  | Ref |  | Ref |  | Ref |  |
| Women | -0.05 | [-0.20,0.10] | -0.08 | [-0.24,0.09] | -0.12 | [-0.27,0.03] | -0.11 | [-0.27,0.06] |
| **Childhood Social Class** |  |  |  |  |  |  |  |  |
| Professional/intermediate | Ref |  |  |  |  |  | Ref |  |
| Non-manual | 0.14 | [-0.10,0.39] |  |  |  |  | 0.11 | [-0.13,0.35] |
| Manual | **0.57** | **[0.38,0.76]** |  |  |  |  | **0.32** | **[0.12,0.52]** |
| Partly skilled/unskilled | **0.62** | **[0.39,0.85]** |  |  |  |  | **0.25** | **[0.00,0.50]** |
| **Adulthood Social Class** |  |  |  |  |  |  |  |  |
| Professional/intermediate |  |  | Ref |  |  |  | Ref |  |
| Non-manual |  |  | **0.2** | **[0.00,0.39]** |  |  | 0.05 | [-0.15,0.25] |
| Manual |  |  | **0.56** | **[0.36,0.76]** |  |  | **0.24** | **[0.02,0.46]** |
| Partly/unskilled |  |  | **0.94** | **[0.71,1.17]** |  |  | **0.54** | **[0.28,0.80]** |
| **Educational Attainment** |  |  |  |  |  |  |  |  |
| University degree |  |  |  |  | Ref |  | Ref |  |
| None |  |  |  |  | **1.01** | **[0.76,1.26]** | **0.62** | **[0.32,0.91]** |
| GCE/School leaving cert |  |  |  |  | **0.3** | **[0.07,0.53]** | 0.14 | [-0.10,0.38] |
| **2015, Age 69** | | | | | | | | |
| **Sex** |  |  |  |  |  |  |  |  |
| Men | Ref |  | Ref |  | Ref |  | Ref |  |
| Women | -0.13 | [-0.33,0.06] | -0.19 | [-0.41,0.03] | -0.23 | [-0.43,-0.04] | **-0.24** | **[-0.46,-0.03]** |
| **Childhood Social Class** |  |  |  |  |  |  |  |  |
| Professional/intermediate | Ref |  |  |  |  |  | Ref |  |
| Non-manual | 0.25 | [-0.07,0.56] |  |  |  |  | 0.18 | [-0.13,0.50] |
| Manual | **0.8** | **[0.53,1.07]** |  |  |  |  | **0.39** | **[0.10,0.68]** |
| Partly/unskilled | **0.98** | **[0.67,1.28]** |  |  |  |  | **0.39** | **[0.06,0.72]** |
| **Adulthood Social Class** |  |  |  |  |  |  |  |  |
| Professional/intermediate |  |  | Ref |  |  |  | Ref |  |
| Non-manual |  |  | 0.36 | [0.08,0.63] |  |  | 0.11 | [-0.17,0.39] |
| Manual |  |  | **0.84** | **[0.55,1.13]** |  |  | **0.33** | **[0.03,0.63]** |
| Partly skilled/unskilled |  |  | **1.45** | **[1.10,1.81]** |  |  | **0.83** | **[0.45,1.20]** |
| **Educational Attainment** |  |  |  |  |  |  |  |  |
| University degree |  |  |  |  | Ref |  | Ref |  |
| None |  |  |  |  | **1.65** | **[1.30,2.01]** | **1.08** | **[0.67,1.49]** |
| GCE/School leaving cert |  |  |  |  | **0.58** | **[0.28,0.88]** | **0.35** | **[0.03,0.67]** |
| ^a^Model adjusted for sex and childhood social class ^b^Model adjusted for sex and adulthood social class^c^ Model adjusted for sex and educational attainment ^d^Model adjusted for sex and all three socioeconomic indicators | | | | | | | | |

**Supplemental Table I. Multimorbidity trajectories and rates in change between adulthood (age 36) and old age (age 69) predicted by linear spline mixed-effects modelling in 3,723 participants from the 1946 MRC National Survey of Health and Development – Models without interactions**

|  | **Model 1**  **Childhood social class** | **Model 2**  **Adulthood social class** | **Model 3**  **Educational level** | **Model 4**  **All socioeconomic indicators** |
| --- | --- | --- | --- | --- |
|  | **β 95% CI** | **β 95% CI** | **β 95% CI** | **β 95% CI** |
| **Spline variables** |  |  |  |  |
| *Change in multimorbidity score per year in each spline variable* |  |  |  |  |
| **Ages 36 to 43 (1982-89)** | **0.08 0.07, 0.09** | **0.08 0.07, 0.09** | **0.08 0.07, 0.09** | **0.08 0.07, 0.09** |
| **Ages 43 to 53 (1989-99)** | **0.06 0.05, 0.07** | **0.06 0.05, 0.07** | **0.06 0.05, 0.07** | **0.06 0.05, 0.07** |
| **Ages 53 to 63 (1999-09)** | **0.07 0.06, 0.08** | **0.07 0.06, 0.07** | **0.07 0.06, 0.07** | **0.07 0.06, 0.07** |
| **Ages 63 to 69 (2009-15)** | **0.19 0.18, 0.20** | **0.19 0.18, 0.20** | **0.19 0.18, 0.20** | **0.19 0.18, 0.20** |
|  |  |  |  |  |
| **Sex** |  |  |  |  |
| Men | **Ref** | **Ref** | **Ref** | **Ref** |
| Women | **0.15 0.09, 0.21** | **0.13 0.06, 0.20** | **0.15 0.09 0.20** | **0.13 0.07, 0.20** |
|  |  |  |  |  |
| **Childhood Social Class** |  |  |  |  |
| Professional/intermediate | **Ref** |  |  | **Ref** |
| Non-manual | 0.03 -0.06, 0.12 |  |  | 0.03 -0.07, 0.12 |
| Manual | 0.06 -0.01, 0.14 |  |  | 0.03 -0.05, 0.10 |
| Partly skilled/unskilled | 0.08 -0.01, 0.16 |  |  | 0.02 -0.08, 0.11 |
|  |  |  |  |  |
| **Adulthood Social Class** |  |  |  |  |
| Professional/intermediate |  | **Ref** |  | **Ref** |
| Non-manual |  | 0.05 -0.03, 0.13 |  | 0.04 -0.04, 0.13 |
| Manual |  | 0.06 -0.02, 0.14 |  | 0.03 -0.06, 0.12 |
| Partly skilled/unskilled |  | **0.21 0.12, 0.30** |  | **0.17 0.07, 0.27** |
|  |  |  |  |  |
| **Educational Attainment** |  |  |  |  |
| University degree |  |  | **Ref** | **Ref** |
| None |  |  | **0.11 0.01, 0.21** | 0.04 -0.09, 0.16 |
| GCE/School leaving certificate |  |  | -0.02 -0.12, 0.07 | -0.05 -0.15, 0.05 |
|  |  |  |  |  |
| **Multimorbidity score at age 36 *(constant)*** | **0.40 0.30, 0.51** | **0.43 0.33, 0.53** | **0.44 0.32, 0.56** | **0.44 0.31, 0.56** |

Text in bold indicate 95% confidence intervals that do not include zero

**Supplemental Figure A. Results from linear regression modelling assessing associations between socioeconomic position and multimorbidity in 3,723 participants from the 1946 MRC National Survey of Health and Development**

**
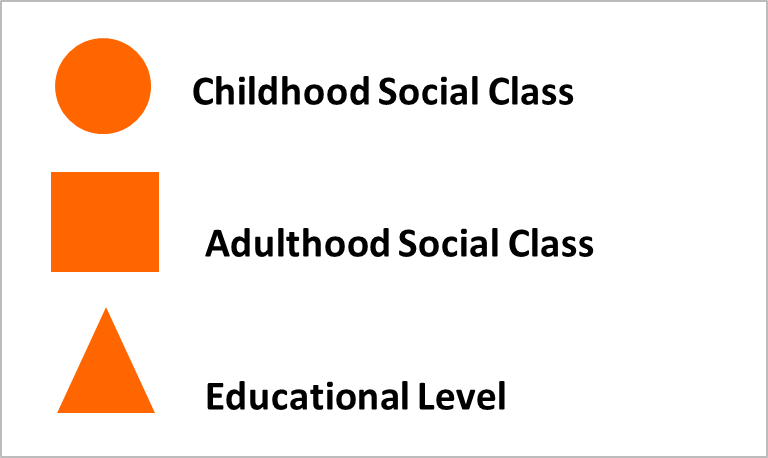

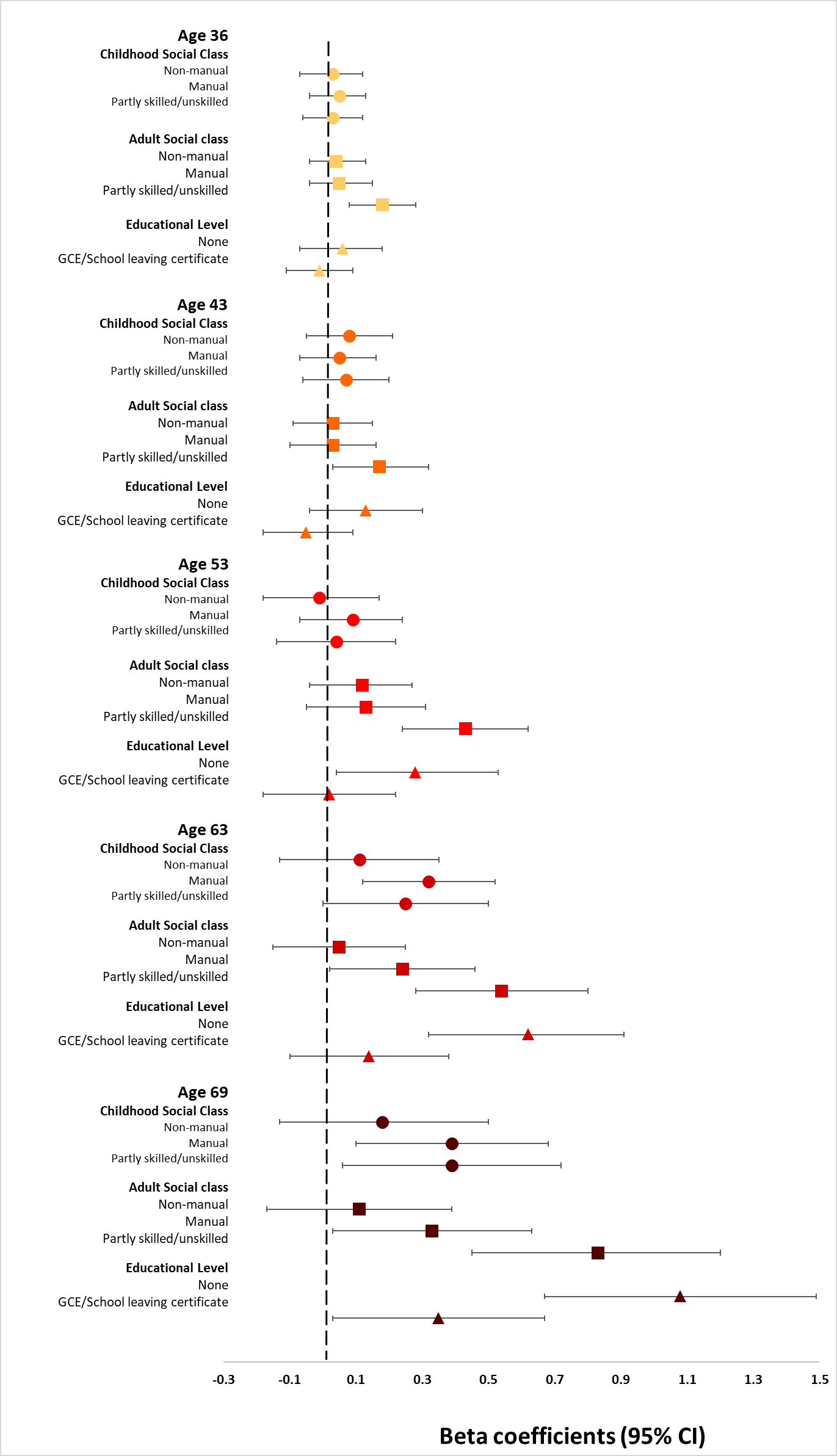
**

**Note: Figure is based on Model 4 in Supplemental Table H.**

**Reference categories:**

*Childhood social class & Adulthood social class:* Professional/intermediate

*Educational level:* University degree

**Supplemental Figure B. Population-based predicted trajectory for multimorbidity from linear spline mixed-effects modelling in 3,723 participants from the 1946 MRC National Survey of Health and Development**

**
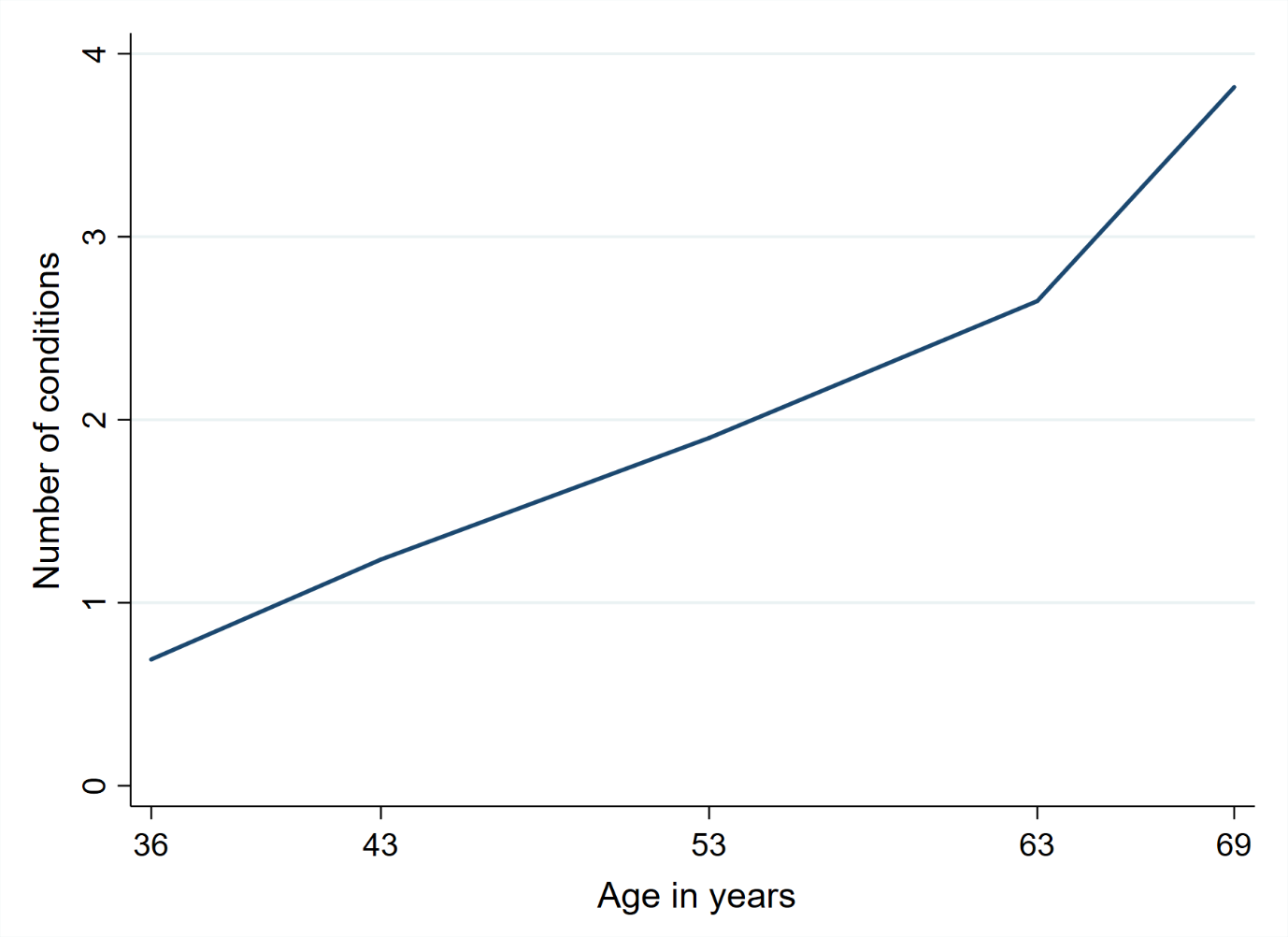
**

**Supplemental Figure C. Population-based predicted trajectories for multimorbidity by sex from linear spline mixed-effects modelling in 3,723 participants from the 1946 MRC National Survey of Health and Development**

**
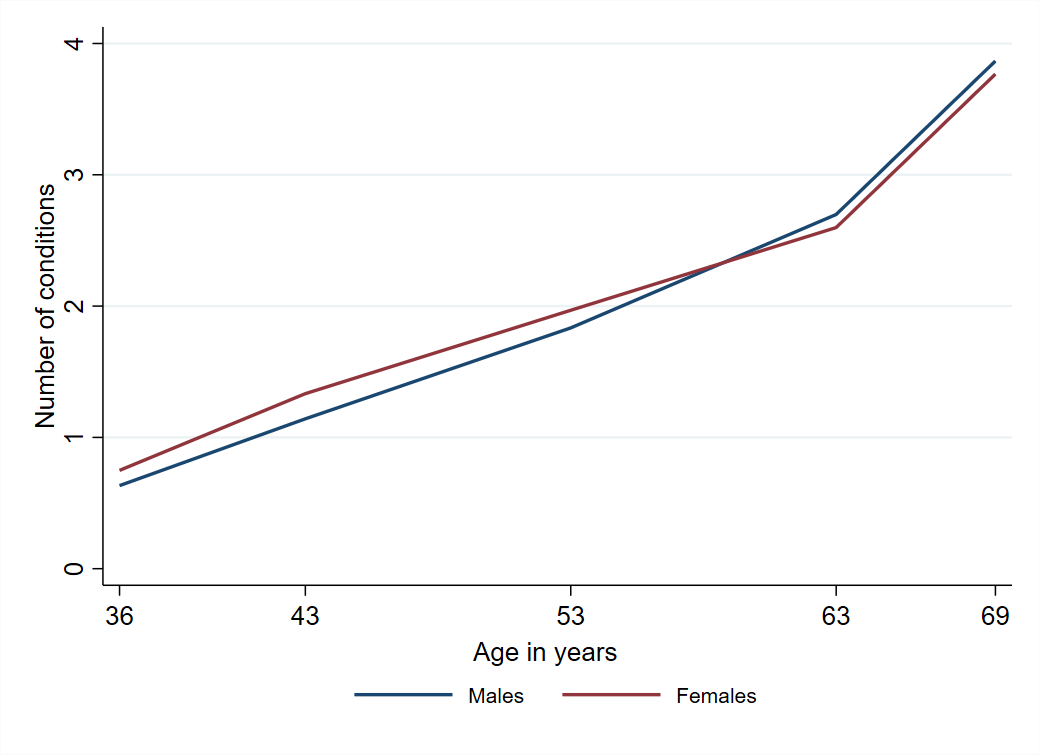
**
